# MGIT-seq for the Identification of Nontuberculous Mycobacteria and Drug Resistance: a Prospective Study

**DOI:** 10.1128/jcm.01626-22

**Published:** 2023-03-22

**Authors:** Kiyoharu Fukushima, Yuki Matsumoto, Takanori Matsuki, Haruko Saito, Daisuke Motooka, Sho Komukai, Eriko Fukui, June Yamuchi, Tadayoshi Nitta, Takayuki Niitsu, Yuko Abe, Hiroshi Nabeshima, Yasuharu Nagahama, Takuro Nii, Kazuyuki Tsujino, Keisuke Miki, Seigo Kitada, Atsushi Kumanogoh, Shizuo Akira, Shota Nakamura, Hiroshi Kida

**Affiliations:** a Department of Respiratory Medicine, National Hospital Organization, Osaka Toneyama Medical Center, Toyonaka, Osaka, Japan; b Department of Respiratory Medicine and Clinical Immunology, Osaka University Graduate School of Medicine, Suita, Osaka, Japan; c Department of Host Defense, Research Institute for Microbial Diseases (RIMD), Osaka University, Osaka, Japan; d Laboratory of Host Defense, World Premier Institute Immunology Frontier Research Center (WPI-IFReC), Osaka University, Osaka, Japan; e Global Center for Medical Engineering and Informatics, Suita, Osaka, Japan; f Department of Infection Metagenomics, Genome Information Research Center, Research Institute for Microbial Diseases (RIMD), Osaka University, Suita, Osaka, Japan; g Department of Clinical Laboratory, National Hospital Organization, Osaka Toneyama Medical Centre, Toyonaka, Osaka, Japan; h Integrated Frontier Research for Medical Science Division, Institute for Open and Transdisciplinary Research Initiatives, Osaka University, Suita, Osaka, Japan; i Department of Biomedical Statistics, Graduate School of Medicine, Osaka University, Suita, Osaka, Japan; j Department of General Thoracic surgery, Osaka University Graduate School of Medicine, Suita, Osaka, Japan; k Kitada Respiratory Clinic, Yao, Osaka, Japan; l Center for Infectious Disease Education and Research, Japan for Infectious Disease Education and Research, Osaka University, Suita, Osaka, Japan; University of Manitoba

**Keywords:** nontuberculous mycobacteria, multilocus sequence typing, macrolide resistance

## Abstract

Because nontuberculous mycobacterial pulmonary disease is a considerable health burden, a simple and clinically applicable analytical protocol enabling the identification of subspecies and drug-resistant disease is required to determine the treatment strategy. We aimed to develop a simplified workflow consisting only of direct sequencing of mycobacterial growth indicator tube cultures (MGIT-seq). In total, 138 patients were prospectively enrolled between April 2021 and May 2022, and culture-positive MGIT broths were subjected to sequencing using MinION, a portable next-generation sequencer. Sequence analysis was conducted to identify species using core genome multilocus sequence typing and to predict macrolide and amikacin (AMK) resistance based on previously reported mutations in *rrl, rrs*, and *erm*(41). The results were compared to clinical tests for species identification and drug susceptibility. A total of 116 patients with positive MGIT cultures were included in the analysis. MGIT-seq yielded 99.1% accuracy in species-level identification and identified 98 isolates (84.5%) at the subspecies level. Macrolide and AMK resistance were detected in 19.4% and 1.9% of Mycobacterium avium complex (MAC) and Mycobacterium abscessus isolates. The predicted macrolide and AMK resistance was consistent with the results of conventional drug susceptibility tests, with specificities of 97.6% and 100.0%, respectively. Direct MGIT-seq has achieved comprehensive identification and drug resistance detection of nontuberculous mycobacteria, which could be applicable to determine the treatment strategy by a single test in clinical practice.

## INTRODUCTION

The incidence of nontuberculous mycobacterial pulmonary disease (NTM-PD) is increasing worldwide; its occurrence rate has surpassed that of Mycobacterium tuberculosis infections in developed countries (1). NTM-PD incidence and prevalence are estimated to be 8.6 to 14.7 per 100,000 person-years and 33 to 65 per 100,000 persons, respectively, in Japan (2–4). Approximately 200 NTM species have been identified, two-thirds of which have been reported to be pathogenic (5–8) and can be further classified into many subspecies that show varied clinical characteristics and therapeutic responses (9–11). The clinical course of NTM-PD is heterogeneous, with some patients remaining stable without the need for treatment and others developing refractory disease needing long-term multidrug combination therapy associated with considerable 5-year mortality (25 to 40%), depending on both bacterial and host factors (12–14). Moreover, the difference in the etiological organism at the subspecies level affects the prognosis (15). Although multiple antimicrobial regimens, including macrolides, have been developed over the last decade, their success rates are unsatisfactory, partly because of the emergence of macrolide resistance (14, 16). Furthermore, amikacin (AMK) is an important therapeutic drug for severe and refractory cases, and its efficacy correlates with the results of drug susceptibility tests (9). Therefore, comprehensive pathogen identification at the subspecies level and detection of macrolide and AMK resistance are crucial for NTM-PD management.

With the advancement of next-generation sequencing technology (NGS), a portable NGS device such as MinION, developed by Oxford Nanopore Technologies (ONT; Oxford, UK), enables the evaluation and analysis of sequencing data in real time (17–19). Additionally, liquid culture systems, such as mycobacterial growth indicator tubes (MGITs), dramatically reduce diagnosis time for mycobacterial infections (20). However, identification methods using NGS are still limited to typical NTM, as with other technologies (21–23). This requires multiple tests to identify NTM and detect drug resistance, which further complicates clinical management. Furthermore, direct sequencing of positive MGIT broth using MinION (MGIT-seq) has only been conducted for M. tuberculosis, and it has never been used for the identification of NTM, which has diverse species and subspecies (24–26).

Here, we developed a simple method that can be used in current microbiological laboratory settings to comprehensively identify NTM at the subspecies level using core genome multilocus sequence typing (cgMLST) and predict macrolide resistance directly from MGIT culture-positive broths using the MinION sequencer.

## MATERIALS AND METHODS

### Study design and patients.

We performed a prospective study to assess the clinical utility of the direct MGIT sequencing technique for comprehensive subspecies-level identification and drug resistance prediction in comparison with standard clinical protocol. We consecutively enrolled patients who had been diagnosed with or suspected of NTM-PD and provided written informed consent to participate between April 2021 and May 2022 in Osaka Toneyama Medical Center.

### Data collection.

Sputum samples submitted for the mycobacterial culture test were inoculated into MGIT broth and cultured using the Bactec MGIT 960 instrument (Becton, Dickinson Cockeysville, MD, USA) (20). Positive MGIT broths (flagged positive by the instrument and acid-fast bacillus [AFB] smear was confirmed positive) using the MGIT 960 instrument were subjected to a transcription-reverse transcription concerted reaction (TRC) for the rapid detection of the M. tuberculosis complex by TRCReady-80 (Tosoh Bioscience, Tokyo, Japan) (27). The detailed methods for TRC are described in the supplemental methods.

### Standard clinical identification protocol.

The standard clinical identification protocol was as follows: positive MGIT broths were examined using TRC for M. avium and M. intracellulare. If the results were negative, matrix-assisted laser desorption ionization–time of flight mass spectrometry (MALDI-TOF MS) was performed. If M. abscessus was identified, multiplex PCR- and chromatographic detection-based identification was performed to distinguish M. abscessus subsp. *abscessus*, M. abscessus subsp. *bolletii*, and M. abscessus subsp. *massiliense* (28). Drug susceptibility tests were performed using a microdilution method. We determined the MIC in Mycobacterium avium complex (MAC) for CLR and AMK after confirming adequate growth of the control over 7 days of incubation in a standard atmosphere at 35°C. In M. abscessus, there were 3 and 14 days of incubation (for detection of both acquired and inducible resistance) for CLR and 3 days of incubation for AMK in a standard atmosphere at 30°C. Here, MALDI-TOF MS was performed on all isolates.

The detailed methods for MALDI-TOF MS, multiplex PCR- and chromatographic detection-based identification, and drug susceptibility tests for CLR and AMK are described in the supplemental materials and methods.

### Breakpoints for CLR and AMK.

The breakpoints for CLR were as follows: (i) for MAC, 8 μg/mL, 16 μg/mL, and 32 μg/mL indicated susceptible, intermediate, and resistant isolates, respectively; and (ii) for M. abscessus, 2 μg/mL, 4 μg/mL, and 8 μg/mL indicated susceptible, intermediate, and resistant isolates, respectively. For both MAC and M. abscessus, isolates with AMK MICs of 16 μg/mL, 32 μg/mL, and ≥64 μg/mL were considered susceptible, intermediate, and resistant, respectively (29–32).

### MGIT-seq and cgMLST analyses.

Genomic DNA was extracted from 250 μL of sediment from MGIT cultures using 0.2 g of glass beads (BioSpec Products, USA) in a 1.5-mL tube. The tube was shaken for 5 min at maximum speed using the Disruptor Genie (Scientific Industries, USA), heated for 5 min at 95°C, and centrifuged for 5 min at 13,000 × *g*. Supernatants (100 μL) were collected for library preparation using the rapid PCR barcoding kit (catalog no. SQK-RPB004; ONT). The libraries were sequenced using the MinION sequencer (catalog no. MIN-101B; ONT) with flow cells R9.4.1 (catalog no. FLO-MIN106D; ONT) for 72 h and base called using guppy (v4.3.4).

The samples were identified through cgMLST using mlstverse software (18). After mapping raw sequencing reads to the reference sequence using minimap2, MLST scores were calculated using mlstverse.Mycobacterium.db. Y.M., D.M., and S.N., who were blinded to the clinical data, performed sequencing analyses.

### Drug susceptibility predictions.

The raw sequencing reads were mapped to the reference sequences of *rrl* (NC_016946.1, 1639789 to 1642895) and *erm*(41) (NZ_CP014955.1, 2353195 to 2353716) using minimap2 2.17, and sequence correction was performed using the same method described above. We checked the mutations in *rrl* in positions 2057 to 2058 in Escherichia coli, which corresponded to positions 2267 to 2268 in *rrl* of the reference sequences.

For *erm*(41), the presence of the gene was determined by enumerating the mapped base positions in the reference. We used 10% of the mean depth as the threshold to check whether each base position was mapped. We concluded that *erm*(41) was truncated if the ratio of the number of mapped bases to its total length was 0.2 to 0.8. To predict macrolide resistance, mutations at positions 2058 and 2059 in *rrl*, encoding 23S rRNA in MAC and M. abscessus and T28C in *erm*(41) in M. abscessus, were predicted to be resistant. The complete form of *erm*(41) was also predicted to be resistant.

Similarly, the mutations at position 1408 in *rrs*, encoding 16S rRNA in MAC and M. abscessus, were checked to predict amikacin resistance.

The detailed methods for other analyses used herein are described in the supplemental materials and methods.

### Ethics approval and consent to participate.

The experimental protocol for data involving human participants followed the Ethical Guidelines of the Japan Ministries of Health and Labor for Medical and Health Research Involving Human Subjects. All the experiments were conducted in accordance with the principles outlined in the Declaration of Helsinki. The ethics board of the National Hospital Organization, Osaka Toneyama Medical Center (TNH-R-2020020), approved this prospective study. Written informed consent was obtained from all the participants at enrollment.

### Data availability.

The data sets supporting the conclusions of this study are included in this article. The data sets generated and analyzed here are available from the corresponding author upon reasonable request. The raw sequencing data supporting the findings of this study have been deposited in the NCBI’s SRA under BioProject PRJDB12894.

## RESULTS

### Study subjects.

**FIG 1:**
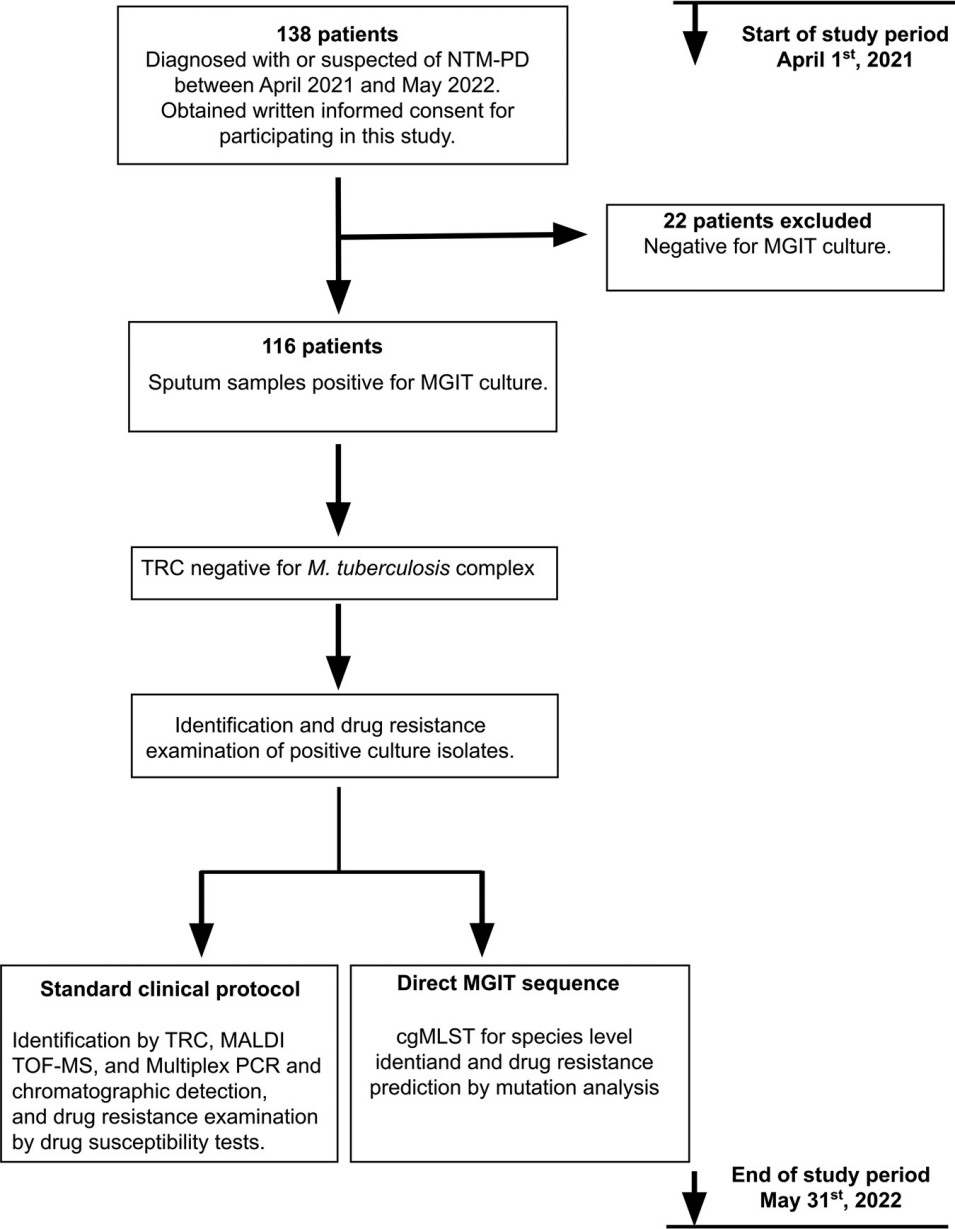
Study design. NTM-PD, nontuberculous mycobacterial pulmonary disease; MGIT, mycobacterial growth indicator tube; TRC, transcription-reverse transcription concerted reaction; MALDI-TOF MS, matrix-assisted laser desorption ionization–time of flight mass spectrometry; cgMLST, core genome multilocus sequence typing.

Initially, 138 patients were enrolled (Fig. 1). Twenty-two patients were excluded from the study because their sputum cultures, analyzed using MGIT broth, were negative for acid-fast bacilli. The final cohort consisted of 116 patients whose clinical characteristics are shown in Table 1. The MGIT broths of all 116 patients were confirmed to be negative for M. tuberculosis using TRC reaction and then directly subjected to sequencing using MinION along with standard clinical identification and drug susceptibility tests.

**TABLE 1:** Clinical characteristics of study population (*n* = 116)^*a*^

Characteristic	Value
No. (%) male	34 (29.3)
Age (mean [SD] [yrs])	71.58 (11.05)
BMI	18.61 (3.227)
Underlying disease (no. [%])	
COPD	2 (1.7)
DM	7 (6.0)
Old TB	6 (5.2)
CRP (mean [SD])	1.343 (3.420)
Radiological features	
Disease type (no. [%])	
NB type	98 (84.5)
FC type	14 (12.1)
Unclassifiable	4 (3.4)
Cavity (no. [%])	40 (34.5)
AFB stain positive (no. [%])	71 (61.2)
Previous isolation of NTM (no. [%])	86 (74.1)

a BMI, body mass index; COPD, chronic obstructive pulmonary disease; DM, diabetes mellitus; Old TB; old tuberculosis; CRP, C-reactive protein; NB, nodular bronchiectasis; FC, fibrocavitary; AFB, acid-fast bacilli; NTM, nontuberculous mycobacteria.

### Mycobacterial identification using the standard protocols versus cgMLST from MGIT-seq.

**FIG 2:**
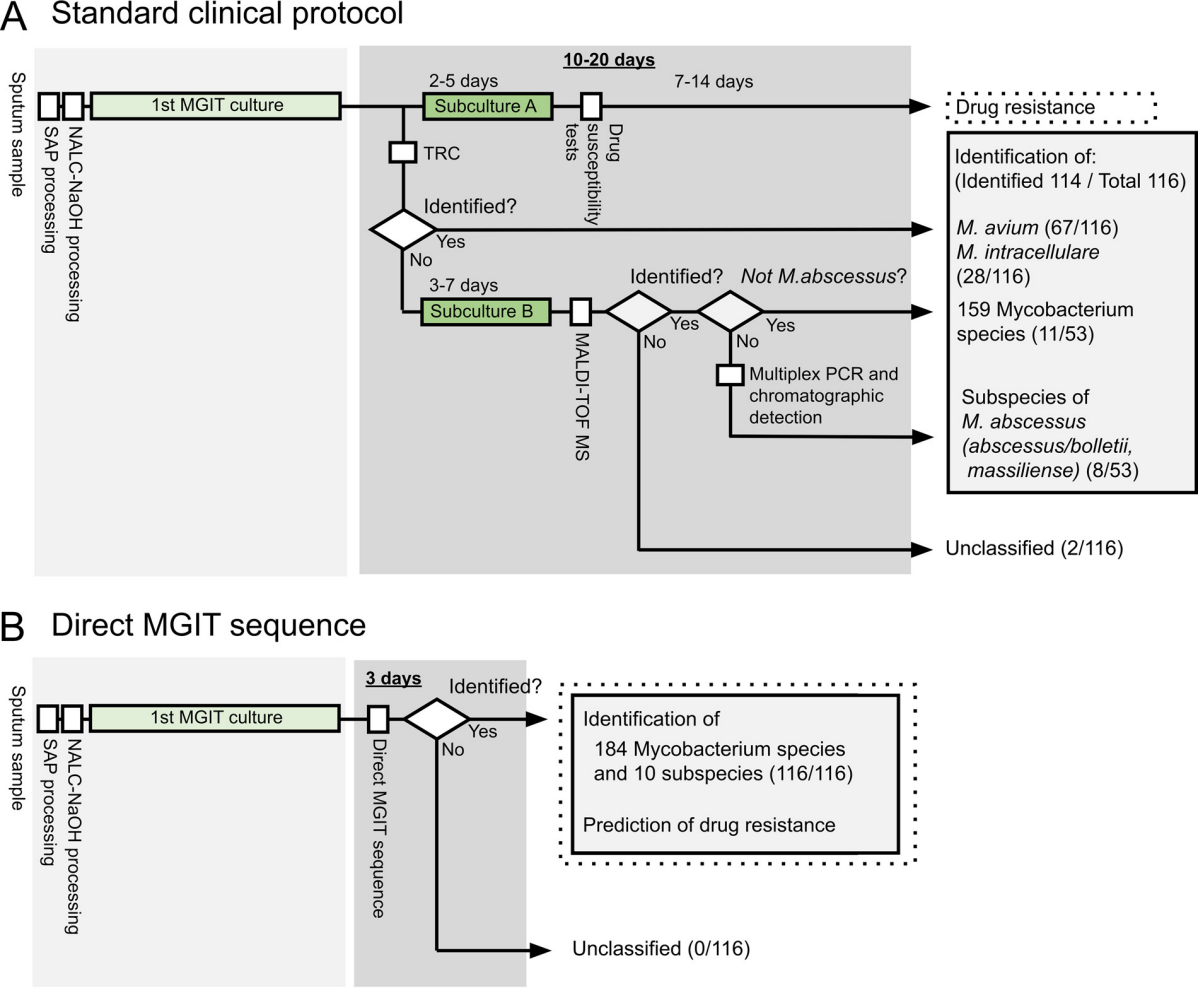
Comparison of workflows for the identification of nontuberculous mycobacteria (NTM) between the standard clinical protocol (A) and direct MGIT sequencing (B). SAP, semialkaline protease; NALC-NaOH, *N*-acetyl-l-cysteine-NaOH; MGIT, mycobacterial growth indicator tube; TRC, transcription-reverse transcription concerted reaction; MALDI-TOF MS, matrix-assisted laser desorption ionization–time of flight mass spectrometry.

The study flow of standard clinical protocols and cgMLST from MGIT-seq are presented in Fig. 2. The results are summarized in Table 2 and Table S1 in the supplemental material. Standard clinical protocols included TRC, MALDI-TOF MS, multiplex PCR, and chromatographic detection. Positive MGIT broths were initially examined using TRC for M. avium and M. intracellulare. Sixty-seven isolates (67/116; 57.8%) were positive for M. avium. Twenty-seven isolates (27/116, 23.3%) were positive for M. intracellulare. The remaining 22 isolates were subjected to MALDI-TOF MS after the subculture on 7H11 agar medium for 3 to 7 days and identified as M. abscessus (*n* = 8), M. kansasii (*n* = 1), M. lentiflavum (*n* = 2), M. peregrinum (*n* = 2), M. avium (*n* = 1), M. paragordonae (*n* = 1), M. gordonae (*n* = 1), M. fortuitum complex (*n* = 2), and M. chelonae (*n* = 1). Three isolates were not identified using MALDI-TOF MS. Eight isolates of M. abscessus were further analyzed using multiplex PCR and chromatographic detection to distinguish M. abscessus subsp. *abscessus,* M. abscessus subsp. *bolletii*, and M. abscessus subsp. *massiliense*. Five isolates were identified as M. abscessus subsp. *massiliense* and three as M. abscessus subsp. *abscessus* and M. abscessus subsp. *bolletii*. In total, these standard clinical protocols were used to classify 113 isolates (97.4%) at the species level and eight isolates (6.9%) at the subspecies level.

**TABLE 2:** Identification results by different identification methods of all 116 patients^*a*^

Identification method and species	No. (%) identified
MGIT-seq, cgMLST	
M. avium subsp. *hominissuis*	68 (58.6)
M. intracellulare subsp. *intracellulare*	25 (21.6)
M. intracellulare subsp. *chimaera*	2 (1.7)
M. abscessus subsp. *abscessus*	3 (2.6)
M. abscessus subsp. *massiliense*	5 (4.3)
M. kansasii	1 (0.9)
M. lentiflavum	2 (1.7)
M. peregrinum	2 (1.7)
M. fortuitum subsp. *fortuitum*	1 (0.9)
M. porcinum	1 (0.9)
M. paragordonae	3 (2.6)
M. gordonae	1 (0.9)
M. chelonae	1 (0.9)
M. szulgai	1 (0.9)
TRC	
M. avium	67 (57.9)
M. intracellulare	27 (23.3)
MALDI-TOF MS	
M. avium	68 (58.6)
M. intracellulare	27 (23.3)
M. abscessus	8 (6.9)
M. kansasii	1 (0.9)
M. lentiflavum	2 (1.7)
M. peregrinum	2 (1.7)
M. fortuitum complex	2 (1.8)
M. paragordonae	1 (0.9)
M. gordonae	1 (0.9)
M. chelonae	1 (0.9)
Unidentified	3 (2.6)
Multiplex PCR and chromatographic detection	
M. abscessus subsp. *abscessus*/M. abscessus subsp. *bolletii*	3 (2.6)
M. abscessus subsp. *massiliense*	5 (4.3)

a MGIT-seq, direct sequence of positive mycobacterial growth indicator tube broth; cgMLST, core genome multilocus sequence typing; TRC, transcription-reverse transcription concerted reaction; MALDI-TOF MS, matrix-assisted laser desorption ionization–time of flight mass spectrometry.

MGIT-seq was used to comprehensively read the DNA extracted from 116 positive MGIT broths. We obtained approximately 1.15 Gbp of total yields corresponding to 263× coverage of the genome size of M. tuberculosis on average per sample using MinION (Table S2). The obtained sequencing data revealed that the fraction of the mycobacterial genome was over 80%, except for one sample (Fig. S1). The sequencing data were then subjected to cgMLST analysis using 184 loci (18). cgMLST analysis identified 68 isolates as M. avium subsp. *hominissuis*, 25 as M. intracellulare subsp. *intracellulare*, two as M. intracellulare subsp. *chimaera*, five as M. abscessus subsp. *massiliense*, three as M. abscessus subsp. *abscessus*, three as M. paragordonae, two as M. lentiflavum, two as M. peregrinum, and one each as M. kansasii, M. chelonae, M. fortuitum subsp. *fortuitum*, M. porcinum, M. szulgai, and M. gordonae.

The MGIT-seq and cgMLST analyses identified all 116 isolates (100%) at the species level and 98 isolates (84.5%) at the subspecies level. All samples showed unique top scores (see Table S3). A discrepancy in cgMLST and the standard clinical protocol results was found in four cases, wherein the standard clinical protocol results showed three unidentified cases (no. 53, 71, and 93), and one case was identified as M. gordonae (no. 85). cg-MLST analysis identified M. gordonae (no. 53), M. szulgai (no. 71), and M. paragordonae (no. 93) in the three unidentified cases. The case of M. gordonae (no. 85) in the standard clinical protocol was identified as M. paragordonae through cg-MLST. These four discrepant cases were further examined using whole-genome comparison through average nucleotide identity (ANI) calculations against 404 assemblies of mycobacterium genome sequences (Table S4). The calculated ANI values indicated these isolates to be M. paragordonae (no. 53) with 88.1% identity to the type strains, M. szulgai (no. 71) with 99.4%, and M. paragordonae (no. 85 and 93) with 87.7 and 87.5%, respectively. The species-level identification accuracy of cgMLST compared to those of standard clinical protocol and ANI in unidentified and discrepant cases as the gold standard was as follows: overall (99.1%), M. avium (100%, 68/68), M. intracellulare (100%, 27/27), M. abscessus (100%, 8/8), M. kansasii (100%, 1/1), M. lentiflavum (100%, 2/2), M. peregrinum (100%, 2/2), M. fortuitum (100%, 1/1), M. porcinum (100%, 1/1), M. paragordonae (75%, 3/4), M. chelonae (100%, 1/1), and M. szulgai (100%, 1/1).

### Drug susceptibility prediction using direct MGIT sequencing.

In total, 103 isolates were subjected to drug susceptibility testing for CLR and AMK (95 MAC and 8 M. abscessus) (Tables 3 and 4 and Tables S5 and S6). Drug susceptibility was determined through the broth microdilution method after the second culture on Ogawa egg medium for rapidly growing mycobacteria, such as M. abscessus, and Middlebrook 7H9 broth for slowly growing mycobacteria, such as MAC. This test detected 21 macrolide-resistant strains, 1 intermediate strain, and 81 susceptible strains. AMK resistance was detected in 2 strains, intermediate in 3 strains, and susceptible in 98 strains.

**TABLE 3:** Efficacy of macrolide resistance prediction by MGIT-seq in comparison of drug susceptibility tests^*a*^

Species (*n* = 103)	Genotype with *rrl* position at:		Data for *erm*(41)		Predicted macrolide susceptibility	No. (%)	Drug susceptibility test	No. (%)
	2058	2059	State	Genotype at position 28				
M. abscessus isolates (*n* = 8)	A	A	Complete	T	R	3 (3.0)	R	3 (3.0)
	A	A	Truncated	T	S	5 (4.9)	S	5 (4.9)
MAC isolates (*n* = 95)	A	A	None		S	77 (74.7)	S	75 (72.8)
							I	1 (1.0)
							R	1 (1.0)
	G	A	None		R	3 (3.0)	R	3 (3.0)
	C	A	None		R	6 (5.8)	R	6 (5.8)
	T	A	None		R	2 (1.9)	R	1 (1.0)
							S	1 (1.0)
	A	G	None		R	3 (3.0)	R	2 (1.9)
							S	1 (1.0)
	A	C	None		R	4 (3.9)	R	4 (3.9)

a MGIT-seq, direct sequence of positive mycobacterial growth indicator tube broth; MAC, Mycobacterium avium complex; R, resistant; I, intermediate; S, susceptible.

**TABLE 4:** Efficacy of amikacin resistance prediction by MGIT-seq in comparison of drug susceptibility tests^*a*^

Species (*n* = 103)	Genotype at *rrs* position 1408	Predicted amikacin susceptibility	No. (%)	Drug susceptibility test	No. (%)
M. abscessus isolates (*n* = 8)	A	S	8 (7.8)	S	8 (7.8)
MAC isolates (*n* = 95)	A	S	94 (91.3)	S	90 (87.4)
				I	3 (2.9)
				R	1 (1.0)
	G	R	1 (1.0)	R	1 (1.0)

a MGIT-seq, direct sequence of positive mycobacterial growth indicator tube broth; MAC, Mycobacterium avium complex; R, resistant; I, intermediate; S, susceptible.

To predict macrolide resistance using MGIT-seq data, we analyzed variants of *rrl* and *erm*(41) genes. The workflow of drug resistance prediction using direct MGIT sequencing is shown in Fig. S2. For detecting mutations in *rrl*, the percent identity reached 99.9% for single nucleotide variants (SNV) and 99.7% for insertions and deletions (indels) using 20× coverage of sequencing data (Fig. S3). Analysis of *rrl* revealed 85 susceptible genotypes of adenine at positions 2058 and 2059 (E. coli numbering) and 18 resistant genotypes at positions 2058 and 2059. Among these resistant genotypes, the following mutation patterns were observed: two A2058T, six A2058C, three A2058G, four A2059C, and three A2059G (Table 3 and Fig. S4A). A discrepancy between drug susceptibility testing (DST) and MGIT-seq results was found in three MAC isolates, two with the susceptible genotype (no. 25) showing higher MICs (MIC of 32), one with resistance genotype A2058T (no. 40) showing a lower MIC (MIC 4), and one with resistance genotype A2059G (no. 57) showing a lower MIC (MIC 2). We then analyzed the *erm*(41) gene, of which the point mutation (T28C) and structural variants were complete or truncated. The truncated form of *erm*(41) was confirmed in five M. abscessus subsp. *massiliense* isolates (Fig. S4B). The T28C mutation in *erm*(41) was not detected in M. abscessus isolates. Diagnostic validities of macrolide resistance predicted using direct MGIT sequencing for phenotypic resistance were as follows: sensitivity, 0.95; specificity, 0.976; positive predictive value, 0.905; and negative predictive value, 0.988.

Analysis of *rrs* revealed 102 susceptible genotypes of adenine at position 2058 (E. coli numbering) and 1 resistant genotype of guanine at position 2058. In summary, diagnostic validities of AMK resistance predicted using direct MGIT sequencing for phenotypic resistance were as follows: sensitivity, 0.500; specificity, 1.00; positive predictive value, 1.00; and negative predictive value, 0.99.

In cases where discrepant results were obtained for macrolide resistance (no. 25, 40, and 57), we reperformed NGS analysis and susceptibility tests for CLR. To match the culture conditions for NGS and susceptibility testing, the same subcultured MGIT specimen was used for NGS and susceptibility testing. The MGIT culture isolates were subcultured in Middlebrook 7H9, and the culture medium was adjusted to a 0.5 McFarland standard with sterile distilled water. Thereafter, samples were subjected to a MinION sequencer and susceptibility testing. Discrepant results were resolved as follows: no. 25 (A2058G, MIC >32), no. 40 (susceptible genotype, MIC 4), and no. 57 (susceptible genotype, MIC 4).

## DISCUSSION

**FIG 3:**
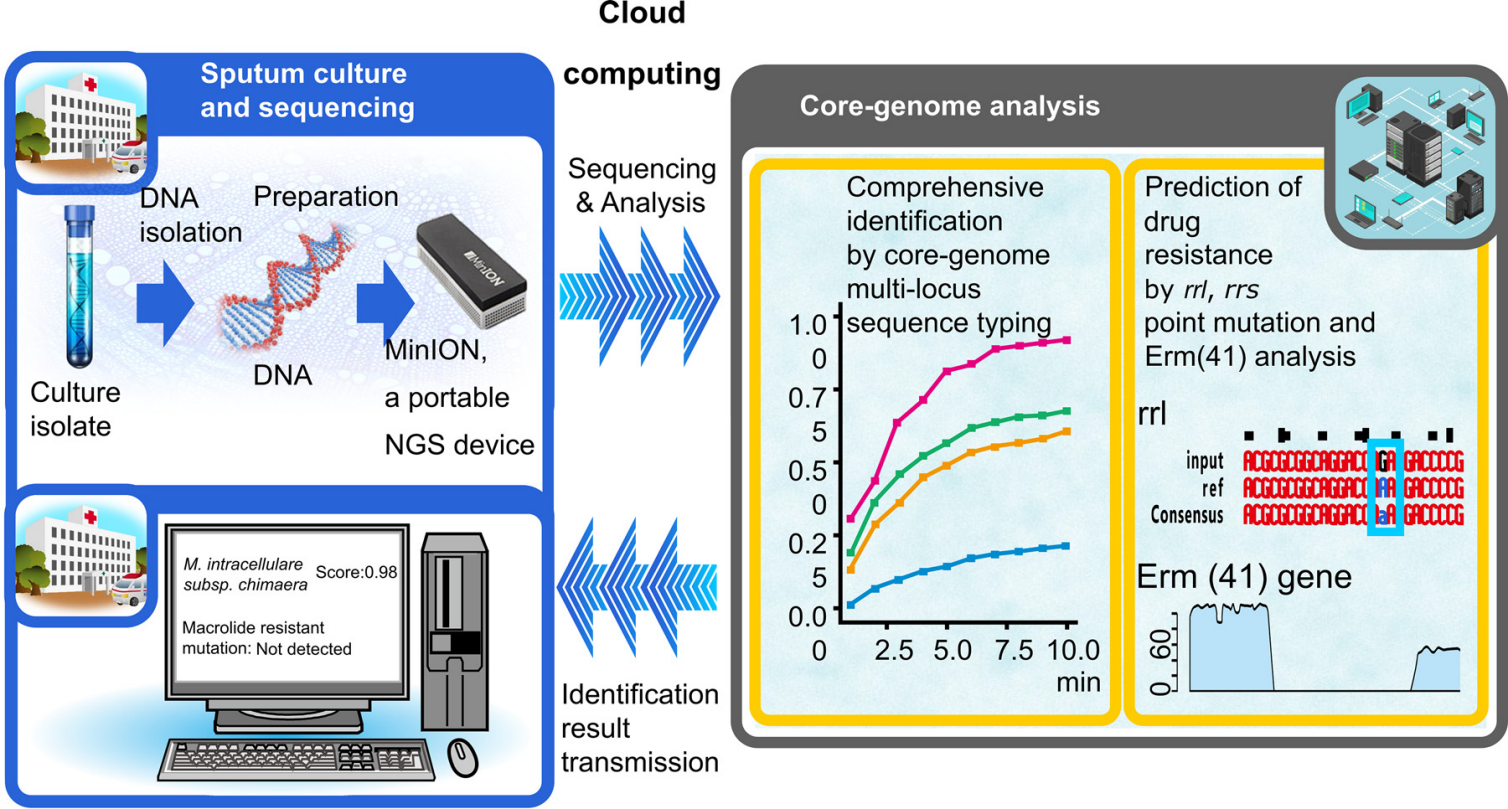
Comprehensive subspecies-level identification and drug resistance prediction using an all-in-one clinical sequencing platform for nontuberculous mycobacteria.

In this prospective study, we showed that comprehensive subspecies-level identification and macrolide resistance prediction in NTM can be performed using a single direct MGIT sequence analysis platform, which can be employed in current clinical laboratory settings. Using this clinical sequencing and analysis method, clinicians dealing with NTM-PD can obtain diagnostic results to determine treatment strategies. As demonstrated in Fig. 2, the turnaround time for mycobacterial identification and susceptibility testing from positive MGIT broths typically ranges from 10 to 20 days. Conversely, the MGIT-seq takes only 3 days from sample preparation to final results. Hence, our workflow shortens the time required for identification and susceptibility testing and simplifies the process and reduces costs. Currently, the process, including basecalling, mapping to the database, score filtering, and variant detection, is already set up to be automatically performed during sequencing. The identified species name and predicted resistance can be viewed on the in-house website. We are working on making it possible to publicly perform this analysis simply by installing client-side software in the future. This analysis requires only a laptop computer and no prior knowledge of bioinformatics. Therefore, our study marks the beginning of an era of clinical sequencing for the comprehensive identification and drug resistance prediction of NTM (Fig. 3).

Typical molecular diagnostic techniques use genetic information to identify NTM species (33). PCR and TRC are performed to detect NTM species using housekeeping genes; however, detectable species are still limited to well-known mycobacteria, such as M. avium and M. intracellulare. In recent years, MS-based methods, such as MALDI-TOF MS, have become more common for comprehensive identification; however, these methods cannot identify mycobacteria at the subspecies level, and the identification results at the species level are not satisfactory, even using subculture for purification. In clinical culture isolates, species-level identification accuracy of MALDI-TOF MS has been reported to be 79.8% to 98.8% (34–41). In this prospective study, three isolates were not identified, and one isolate was misidentified through MALDI-TOF MS. Overall identification accuracy of MALDI-TOF-MS was 96.6%. However, two isolates were identified only at the complex level (M. fortuitum complex). Novel species belonging to the M. gordonae complex have been reported in succession in recent years, and it is possible that there are more unknown species belonging to the M. gordonae clade (42–44). The ANI results indicated that three strains (no. 53, 85, and 93) isolated here were possibly independent of the M. gordonae clade because they showed ANI values <95% (45). As current taxonomic classification relies on genomic aspects, approaches based on genetic information may help unveil the diversity of M. paragordonae species and clarify the overall picture.

Identifying major NTM species with sufficient accuracy for therapeutic decision-making is important for clinical applications. Few prospective studies have examined the applicability of the direct sequencing approach in clinical settings for the comprehensive identification of NTM species. Pankhurst et al. performed a prospective study of mycobacterial diagnosis with whole-genome sequencing involving 177 NTM isolates; however, typical NTM such as MAC, M. abscessus, and M. fortuitum were identified only at the complex level, which was not sufficient for determination of treatment strategies (22). Next, Quan et al. performed the largest prospective study to evaluate whole-genome sequencing for mycobacterial species identification. They examined 1,201 NTM isolates; while overall species-level identification accuracy was 88.3%, it was only 23% for rare species due to the insufficient database, which includes only 40 NTM species (23). More recently, He et al. prospectively analyzed the accuracy of target capture sequencing of 30 clinical samples in M. tuberculosis and NTM identification (21). They showed that sequencing and identification of mycobacteria using clinical samples could be done with NGS but is limited to species-level identification using the database consisting of 32 NTM species, which is insufficient to determine treatment options (9). We performed a single direct MGIT sequence using the cgMLST database containing 175 NTM species and identified NTM species with high accuracy of 99.1% (115/116), including both common and rare NTM species.

Most conventional methods must first identify the species and then classify them into subspecies. There are few methods that can identify species and subspecies simultaneously. In clinical practice, if subspecies-level information is required for treatment selection, additional tests for subspecies identification are needed as described in Fig. 2; thus, in such cases, it can take 20 days from culture-positive indications to final identification and drug susceptibility results (28, 31, 46, 47). Furthermore, for facilities that do not have the equipment for MALDI-TOF MS, it could take more than 1 month from culture-positive indications to final results. The Accuprobe assay, which is a rapid identification test based on the DNA probe method, is no longer available for direct identification of major mycobacterial species such as MAC and M. tuberculosis, and the more sensitive PCR and TRC methods have become mainstream. Recently, PCR and chromatographic detection-based identification assays and subspecies-specific PCR assays have been developed to distinguish between M. abscessus subspecies (28, 48). A line probe assay consisting of PCR and Southern blot hybridization with the appropriate probes, such as the GenoType NTM-DR (Hain Lifescience, Nehren, Germany), can detect several NTM species, such as M. avium, M. intracellulare, M. chelonae, and M. abscessus, and can differentiate subspecies of M. intracellulare and M. abscessus (49, 50). In our study, the clinically important species, which are further classified into several subspecies, such as M. intracellulare and M. abscessus, were detected. The percentage of successful subspecies identification was improved from 6.9% of the clinical standard methods to 84.5%. The method established here simplifies the complicated flow of testing and provides subspecies-level identification.

Macrolides, such as CLR and azithromycin, are the key drugs used to treat MAC-PD, and the results of drug susceptibility tests are correlated with clinical effects. Macrolide resistance is associated with poor treatment outcomes and mortality (49, 51). Therefore, the prevention and early detection of macrolide resistance are particularly important. The primary factors resulting in acquired macrolide resistance are point mutations at positions 2058 and 2059 in domain V of *rrl*. In M. abscessus, induction of the *erm*(41) gene is associated with inducible macrolide resistance, and the T28C mutation in *erm*(41), which was not detected in our study, is associated with acquired resistance. The truncated form of *erm*(41) in M. abscessus subsp. *massiliense* results in macrolide susceptibility. Among 17 MAC-PD cases that showed resistance to macrolides, point mutations at positions 2058 and 2059 were detected in all cases. These results are consistent with the findings of previous studies showing point mutations at these positions in 80% to 100% of macrolide-resistant MAC isolates (49, 51, 52). Considering that macrolide-resistant cases are rare before initial treatment (12, 14, 52), it might be appropriate to consider the start of treatment based on drug resistance prediction, especially in cases of progressive disease. Additionally, AMK is an important therapeutic drug for severe and refractory cases, and its efficacy correlates with the results of drug susceptibility tests (9). Mutations in *rrs* that are responsible for resistance to AMK predominantly occur at position 1408 in both MAC and M. abscessus (53, 54). In our investigation, of the two AMK-resistant isolates, one possessed the A1408G mutation and exhibited the corresponding MIC of >256 μg/mL. Conversely, the A1408G mutation in *rrs* was not present in the isolate with an MIC of 64 μg/mL, consistent with prior studies (55–58). Almost all MAC isolates with MICs of >64 μg/mL have been reported to harbor mutations in *rrs* (57, 59), and high AMK MICs (>64 μg/mL) are linked to treatment failure (59, 60). Therefore, clinical therapeutic efficacy for isolates with AMK MICs of 64 μg/mL requires further validation (55).

In the method established in this study, genomic DNA was amplified and sequenced after extraction using bead shaking. The preparation of a barcoded library from a small amount of genomic DNA can be completed in 15 min of hands-on preparation using equipment in a typical bacterial laboratory. In addition, the total cost of MGIT-seq per sample without flow cell reuse was $59.1 (see Table S7 in the supplemental material). In contrast, a standard clinical protocol consisting of TRC, MALDI-TOF MS, multiplex PCR, and chromatographic detection exceeds more than $100 in a clinical situation in Japan, and the cost of drug susceptibility tests is $27.8. Considering the accuracy and comprehensiveness, MGIT-seq is affordable in clinical practice. A recent study proposed a direct M. tuberculosis detection method using MinION (26). Furthermore, Deeplex Myc-TB (GenoScreen, Lille, France), based on PCR-based targeted deep sequencing and automated data analysis via web application, enabled direct identification and prediction of drug resistance of M. tuberculosis in clinical samples (61–64). Improvement of analysis techniques will allow accurate identification of the entire mycobacterial family directly from sputum samples.

This study has several limitations. First, there were relatively few M. abscessus and rarer NTM species isolates. While a sufficient number of MAC isolates were included, it would be beneficial if more M. abscessus isolates are studied. The same is true for non-MAC and non-M. abscessus species for the identification portion of the study. Second, this study was conducted in a single referral center in Japan; wider applicability should be examined in future multicenter studies.

In conclusion, comprehensive subspecies-level identification and macrolide resistance prediction in NTM could be completed in one-shot direct sequencing from MGIT culture, which is applicable to current clinical laboratory settings.
