# Supplementary material for: MGIT-seq for the Identification of Nontuberculous Mycobacteria and Drug Resistance: a Prospective Study
Source: J Clin Microbiol. 2023 Mar 22;61(4):e01626-22. doi: 10.1128/jcm.01626-22 (PMC10117113; doi:10.1128/jcm.01626-22)
Supplement: Supplemental file 1 — Supplemental material. Download jcm.01626-22-s0001.pdf, PDF file, 0.9 MB [file jcm.01626-22-s0001.pdf]

Supporting Online Material for

MGIT-seq for the Identification of Nontuberculous Mycobacteria and Drug Resistance: A prospective study

Kiyoharu Fukushima, Yuki Matsumoto, Takanori Matsuki, Haruko Saito, Daisuke Motooka, Sho Komukai, Eriko Fukui, June Yamuchi, Tadayoshi Nitta, Takayuki Niitsu, Yuko Abe, Hiroshi Nabeshima, Yasuharu Nagahama, Takuro Nii, Kazuyuki Tsujino, Keisuke Miki, Seigo Kitada, Atsushi Kumanogoh, Shizuo Akirac, Shota Nakamura & Hiroshi Kida

This PDF file includes

Supplementary Methods
Figures. S1 to S4
Tables. S1 to S7

Supplementary Methods

Sample size calculation

The optimal sample size of this study was calculated as follows (1): 100% species level identification is expected using the MGIT-seq, and the performance of the standard clinical protocol is assumed to be 90% (2-4). When two methods are applied to each sample, the rate of identification by MGIT-seq alone and not by the standard clinical protocol is set to 10%, whereas the rate of identification by the standard clinical protocol alone is set to 0.5%. Therefore, assuming a type 1 error of 0.05 and a power of 80%, the required number of cases was 118. Considering the possibility of a 10% dropout, the required sample size for this study was 132.

TRC

M. tuberculosis, *M. avium*, and *M. intracellulare* were differentiated by TRCReady-80 (Tosoh Bioscience, Tokyo, Japan) based on the TRC, which identified each species.

MALDI-TOF MS

Identification of NTM species by MALDI-TOF MS was performed in LSI Medience Corporation (Tokyo, Japan). Positive MGIT broths were subcultured on 7H11 agar medium for 3–7 days. Isolated colonies were transferred into 300 µL deionized water using a 1-µL inoculation loop, and mixed thoroughly. Following heat inactivation at 95°C for 30 min, 900 µL absolute ethanol was added. After centrifugation, the supernatant was removed completely and the pellet was air-dried. Silica beads (0.5 mm diameter) and 20 µL acetonitrile were added to the reaction tube, which was mixed using a vortex mixer for 5 min. Twenty microliters of 70% formic acid was added and mixed well in the tube. After centrifugation, 1 µL supernatant was transferred to a MALDI target plate. Once the sample was dry, 1 µL Bruker HCCA Matrix Solution (Bruker Daltonics GmbH & Co. KG) was applied. Measurement was performed by MALDI Biotyper system with MBT Compass software. The Bruker bacterial test standard was used for calibration. Identification was performed by MALDI Biotyper system. Spectra were acquired using microflex LT/SH with flexControl version 3.4 and MBT Compass version 4.1 with Mycobacteria module. Mycobacteria library 5.0 containing 912 MSPs was used for bacterial identification. Any samples that did not generate a spectrum were re-extracted. Using the Mycobacteria module, the threshold score for high confidence identification was ≥1.8,

and a score of 1.6–1.8 was considered to be low confidence identification. A score <1.6 indicated no reliable identification. Unidentified NTM species by MALDI-TOF MS were further analyzed by the AccuProbe (Gen-Probe Inc., San Diego, CA, USA).

Multiplex PCR- and chromatographic-detection-based identification

M. abscessus subsp. *Abscessus/bolletii* and *M. abscessus* subsp. *massiliense* were differentiated by Kaneka Nucleic Acid Chromatography Rapidly Growing Mycobacteria (RGM) Identification Kit, which is based on multiplex PCR and chromatographic detection (Kaneka, Hyogo, Japan).

Drug susceptibility testing

Drug susceptibility tests for CLR were performed after MAC and *M. abscessus* were isolated. Macrolide susceptibility was determined through broth microdilution for CLR, BrothMIC NTM for MAC, and BrothMIC RGM for *M. abscessus* (Kyokuto Pharmaceutical Industrial Co. Ltd.). After confirming adequate growth of the control over 7 days of incubation in a standard atmosphere at 35°C in MAC,

and 3 and 14 days of incubation (for detection of both acquired and inducible resistance) in a standard atmosphere at 30°C in *M. abscessus*, we determined the MIC. Drug susceptibility tests for AMK were performed using the broth microdilution method according to Clinical and Laboratory Standards Institute M24-3 guidelines (5). The culture was transferred to Middlebrook 7H9 broth and vortexed. We adjusted the culture medium to a 0.5 McFarland standard with sterile distilled water; thereafter, we added 60 μL of the 0.5 McFarland suspension to a 12mL of Cation-Adjusted Mueller-Hinton Broth (Kyokuto Pharmaceutical Industrial Co. Ltd., https://www.kyokutoseiyaku.co.jp) with (slowly growing mycobacteria) and without (rapidly growing mycobacteria) 5% OADC enrichment and dispensed 100 μL of this solution into each well of the panel. After confirming adequate growth of the control over 7 days of incubation in a standard atmosphere at 35°C in slowly growing mycobacteria and 3 days of incubation in a standard atmosphere at 30°C in rapidly growing mycobacteria, we determined the MIC.

Calculation and species identification using average nucleotide identity (ANI) value

The sequencing reads were assembled using flye 2.9 with default options. The assembled sequences were evaluated for the average

nucleotide identity (ANI) using FastANI with "--fragLen 1000" option against 404 assemblies of Mycobacterium comprising 214 identified spp., 13 sub-spp., and 177 not identified spp.

Accuracy of mutation detection by MGIT-seq

The raw sequencing reads of No. 52 were assembled using flye 2.9 and draft genome was corrected by racon 1.4.2 and medaka 1.5.0. To generate the data for correction, we randomly sampled subset sequences of 5, 10, 15, 20, 25, 50, 100, 150, 200, 400 Mb of sequencing data from the raw sequencing reads. These amounts were corresponding to 1X, 2X, 3X, 4X, 5X, 10X, 20X, 30X, 40X, 80X coverage of 5 Mb, typical size of bacterial genome, respectively. The sampled sequences were mapped to the draft genome using minimap2 2.17, and variant call was performed using bcftools and samtools 1.11 with default parameters. The percent identity to the reference sequence was calculated using blastn 2.9.0.

Costs for MGIT-seq

The cost per sample for MinION sequence was calculated by taking into account flow cell costs, library preparation and sequencing kits, and also includes all other costs per sample preparation and sequencing

Supplementary references (1-5)

1. Connor RJ. 1987. Sample size for testing differences in proportions for the paired-sample design. Biometrics 43:207-11.

2. Cao Y, Wang L, Ma P, Fan W, Gu B, Ju S. 2018. Accuracy of matrix-assisted laser desorption ionization-time of flight mass spectrometry for identification of mycobacteria: a systematic review and meta-analysis. Sci Rep 8:4131.

3. Yoshida M, Sano S, Chien JY, Fukano H, Suzuki M, Asakura T, Morimoto K, Murase Y, Miyamoto S, Kurashima A, Hasegawa N, Hsueh PR, Mitarai S, Ato M, Hoshino Y. 2021. A novel DNA chromatography method to discriminate Mycobacterium abscessus subspecies and macrolide susceptibility. EBioMedicine 64:103187.

4. Hida Y, Hisada K, Shimada A, Yamashita M, Kimura H, Yoshida H, Iwasaki H, Iwano M. 2012. Rapid detection of the Mycobacterium tuberculosis complex by use of quenching probe PCR (geneCube). J Clin Microbiol 50:3604-8.

5. Woods GL. 2018. Performance standards for susceptibility testing of mycobacteria, Nocardia spp, and other aerobic actinomycetes, 1st ed CLSI document no M62. Clinical and Laboratory Standards Institute, Wayne (PA).

Supplementary Figures

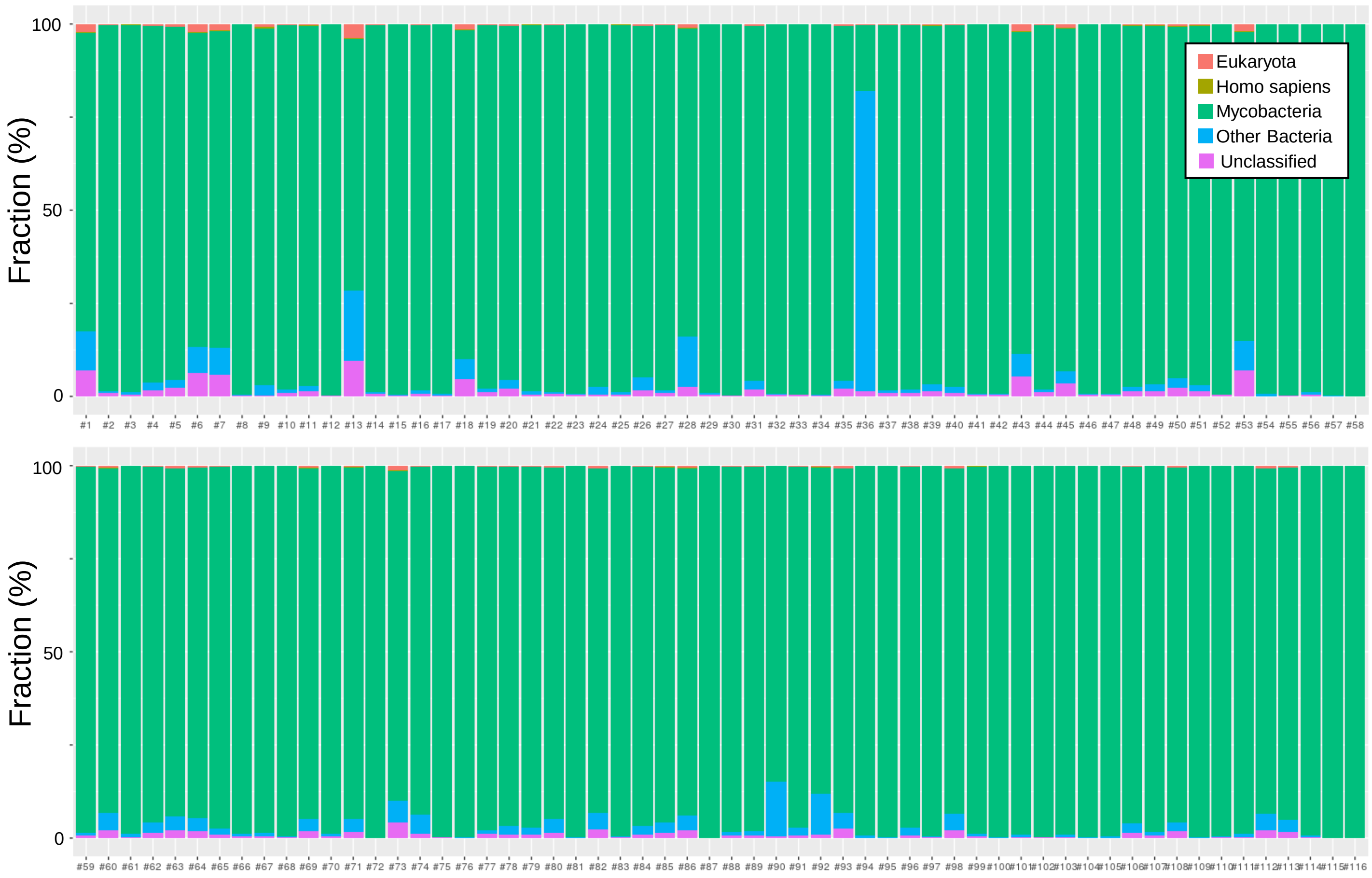

Fig S1. Metagenomics of direct MGIT sequence. Microbiome composition of each sample.

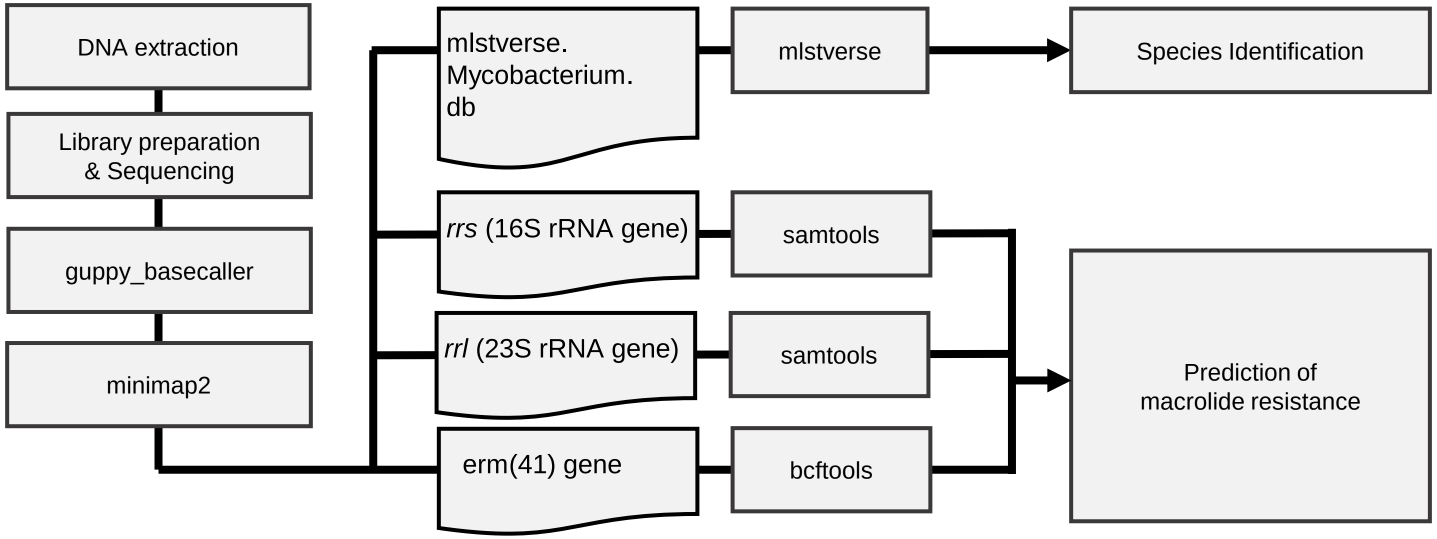

Fig S2. The workflow of sequence analysis.

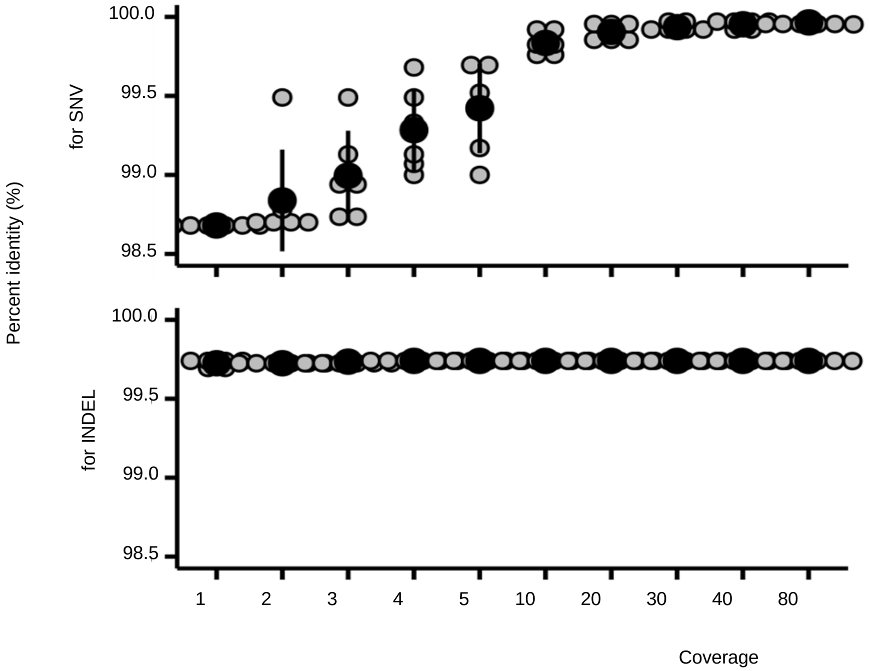

Fig S3. The validity of sequence accuracy of direct MGIT sequence. The percent identity for SNV (top) and INDEL (bottom) were shown with random sampling data (n=6, each; gray) and their average (black). Error bar indicates sd of random sampling.

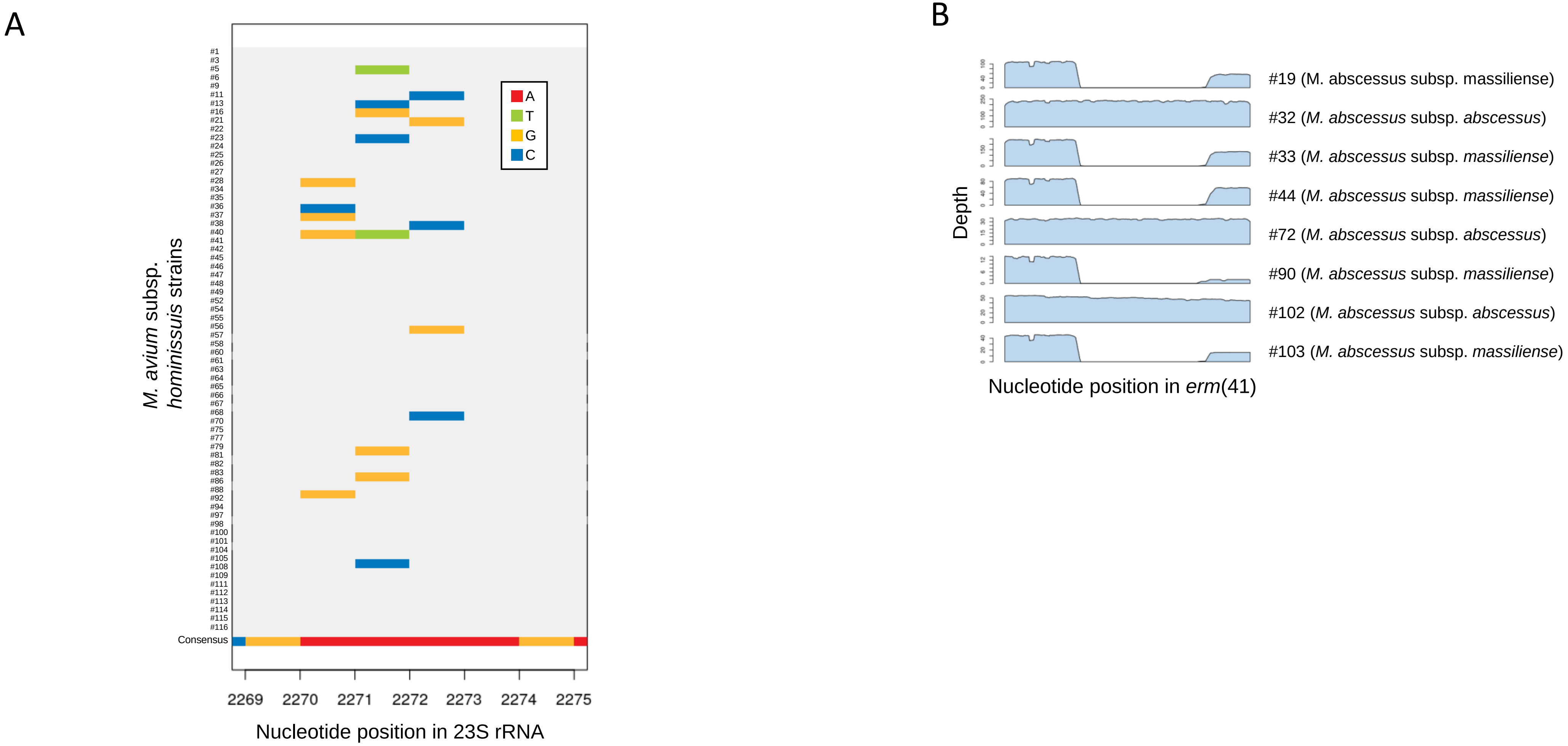

Fig S4. The species identification and drug resistance prediction. A) Macrolide resistance prediction on *rrl* gene. Sixty eight strains of *M. avium* subsp. *hominissuis* were shown. The view of 2269-2275 nucleotide positions in *M. avium* subsp. *hominissuis* (corresponding to 2056-2062 in *E. coli* numbering) were shown. The filled color on each bases indicates the mutation from the consensus base to A (red), to T (green), to G (yellow), and to C (blue), respectively. B) Visualization of *erm*(41) gene status. The depth on each nucleotide position of *erm*(41) gene were shown. *M. avium*, *mycobacterium avium*; subsp, subspecies.

Supplementary Tables

Table S1. Full identification results by different identification methods.

	TRC	MALDI-TOF MS	Multiplex PCR and chromatographic detection	*MLSTverse*
No	species	species	species and subspecies	species and subspecies
1	*M. avium*	*M. avium*		*M. avium* subsp. *hominissuis*
2	*M. intracellulare*	*M. intracellulare*		*M. intracellulare* subsp. *intracellulare*
3	*M. avium*	*M. avium*		*M. avium* subsp. *hominissuis*
4	*M. intracellulare*	*M. intracellulare*		*M. intracellulare* subsp. *intracellulare*
5	*M. avium*	*M. avium*		*M. avium* subsp. *hominissuis*
6	*M. avium*	*M. avium*		*M. avium* subsp. *hominissuis*
7	*M. intracellulare*	*M. intracellulare*		*M. intracellulare* subsp. *intracellulare*
8	*M. intracellulare*	*M. intracellulare*		*M. intracellulare* subsp. *chimaera*
9	*M. avium*	*M. avium*		*M. avium* subsp. *hominissuis*
10	*M. intracellulare*	*M. intracellulare*		*M. intracellulare* subsp. *chimaera*
11	*M. avium*	*M. avium*		*M. avium* subsp. *hominissuis*
12	*M. intracellulare*	*M. intracellulare*		*M. intracellulare* subsp. *intracellulare*
13	*M. avium*	*M. avium*		*M. avium* subsp. *hominissuis*
14	*M. intracellulare*	*M. intracellulare*		*M. intracellulare* subsp. *intracellulare*
15	*M. intracellulare*	*M. intracellulare*		*M. intracellulare* subsp. *intracellulare*

16	*M. avium*	*M. avium*		*M. avium* subsp. *hominissuis*
17		*M. kansasii*		*M. kansasii*
18		*M.lentiflavum*		*M. lentiflavum*
19		*M. abscessus*	*M. massiliense*	*M. abscessus* subsp. *massiliense*
20	*M. intracellulare*	*M. intracellulare*		*M. intracellulare* subsp. *intracellulare*
21	*M. avium*	*M. avium*		*M. avium* subsp. *hominissuis*
22	*M. avium*	*M. avium*		*M. avium* subsp. *hominissuis*
23	*M. avium*	*M. avium*		*M. avium* subsp. *hominissuis*
24	*M. avium*	*M. avium*		*M. avium* subsp. *hominissuis*
25	*M. avium*	*M. avium*		*M. avium* subsp. *hominissuis*
26	*M. avium*	*M. avium*		*M. avium* subsp. *hominissuis*
27	*M. avium*	*M. avium*		*M. avium* subsp. *hominissuis*
28	*M. avium*	*M. avium*		*M. avium* subsp. *hominissuis*
29	*M. intracellulare*	*M. intracellulare*		*M. intracellulare* subsp. *intracellulare*
30	*M. intracellulare*	*M. intracellulare*		*M. intracellulare* subsp. *intracellulare*
31	*M. intracellulare*	*M. intracellulare*		*M. intracellulare* subsp. *intracellulare*
32		*M. abscessus*	*M. abscessus/bolletii*	*M. abscessus* subsp. *abscessus*
33		*M. abscessus*	*M. massiliense*	*M. abscessus* subsp. *massiliense*
34	*M. avium*	*M. avium*		*M. avium* subsp. *hominissuis*
35	*M. avium*	*M. avium*		*M. avium* subsp. *hominissuis*

36	*M. avium*	*M. avium*		*M. avium* subsp. *hominissuis*
37	*M. avium*	*M. avium*		*M. avium* subsp. *hominissuis*
38	*M. avium*	*M. avium*		*M. avium* subsp. *hominissuis*
39	*M. intracellulare*	*M. intracellulare*		*M. intracellulare* subsp. *intracellulare*
40	*M. avium*	*M. avium*		*M. avium* subsp. *hominissuis*
41	*M. avium*	*M. avium*		*M. avium* subsp. *hominissuis*
42	*M. avium*	*M. avium*		*M. avium* subsp. *hominissuis*
43		*M. paragordonae*		*M. paragordonae*
44		*M. abscessus*	*M. massiliense*	*M. abscessus* subsp. *massiliense*
45	*M. avium*	*M. avium*		*M. avium* subsp. *hominissuis*
46	*M. avium*	*M. avium*		*M. avium* subsp. *hominissuis*
47	*M. avium*	*M. avium*		*M. avium* subsp. *hominissuis*
48	*M. avium*	*M. avium*		*M. avium* subsp. *hominissuis*
49	*M. avium*	*M. avium*		*M. avium* subsp. *hominissuis*
50	*M. intracellulare*	*M. intracellulare*		*M. intracellulare* subsp. *intracellulare*
51	*M. intracellulare*	*M. intracellulare*		*M. intracellulare* subsp. *intracellulare*
52	*M. avium*	*M. avium*		*M. avium* subsp. *hominissuis*
53		Unidenfitied		*M. gordonae*
54	*M. avium*	*M. avium*		*M. avium* subsp. *hominissuis*
55	*M. avium*	*M. avium*		*M. avium* subsp. *hominissuis*
56	*M. avium*	*M. avium*		*M. avium* subsp. *hominissuis*
57	*M. avium*	*M. avium*		*M. avium* subsp. *hominissuis*

58	*M. avium*	*M. avium*		*M. avium* subsp. *hominissuis*
59	*M. intracellulare*	*M. intracellulare*		*M. intracellulare* subsp. *intracellulare*
60	*M. avium*	*M. avium*		*M. avium* subsp. *hominissuis*
61	*M. avium*	*M. avium*		*M. avium* subsp. *hominissuis*
62		*M. lentiflavum*		*M. lentiflavum*
63	*M. avium*	*M. avium*		*M. avium* subsp. *hominissuis*
64	*M. avium*	*M. avium*		*M. avium* subsp. *hominissuis*
65	*M. avium*	*M. avium*		*M. avium* subsp. *hominissuis*
66	*M. avium*	*M. avium*		*M. avium* subsp. *hominissuis*
67		*M. avium*		*M. avium* subsp. *hominissuis*
68	*M. avium*	*M. avium*		*M. avium* subsp. *hominissuis*
69	*M. intracellulare*	*M. fortuitum complex*		*M. porcinum*
70	*M. avium*	*M. avium*		*M. avium* subsp. *hominissuis*
71		Unidentified		*M. szulgai*
72		*M. abscessus*	*M. abscessus/bolletii*	*M. abscessus* subsp. *abscessus*
73		*M. fortuitum complex*		*M. fortuitum* subsp. *fortuitum*
74		*M. peregrinum*		*M. peregrinum*
75	*M. avium*	*M. avium*		*M. avium* subsp. *hominissuis*
76	*M. intracellulare*	*M. intracellulare*		*M. intracellulare* subsp. *intracellulare*
77	*M. avium*	*M. avium*		*M. avium* subsp. *hominissuis*
78	*M. intracellulare*	*M. intracellulare*		*M. intracellulare* subsp. *intracellulare*
79	*M. avium*	*M. avium*		*M. avium* subsp. *hominissuis*

80	*M. intracellulare*	*M. intracellulare*		*M. intracellulare* subsp. *intracellulare*
81	*M. avium*	*M. avium*		*M. avium* subsp. *hominissuis*
82	*M. avium*	*M. avium*		*M. avium* subsp. *hominissuis*
83	*M. avium*	*M. avium*		*M. avium* subsp. *hominissuis*
84	*M. intracellulare*	*M. intracellulare*		*M. intracellulare* subsp. *intracellulare*
85		*M. gordonae*		*M. paragordonae*
86	*M. avium*	*M. avium*		*M. avium* subsp. *hominissuis*
87	*M. intracellulare*	*M. intracellulare*		*M. intracellulare* subsp. *intracellulare*
88	*M. avium*	*M. avium*		*M. avium* subsp. *hominissuis*
89	*M. intracellulare*	*M. intracellulare*	*M. abscessus/bolletii*	*M. intracellulare* subsp. *intracellulare*
90		*M. abscessus*	*M. masilliense*	*M. abscessus* subsp. *massiliense*
91	*M. intracellulare*	*M. intracellulare*		*M. intracellulare* subsp. *intracellulare*
92	*M. avium*	*M. avium*		*M. avium* subsp. *hominissuis*
93		*Unidentified*		*M. paragordonae*
94	*M. avium*	*M. avium*		*M. avium* subsp. *hominissuis*
95	*M. intracellulare*	*M. intracellulare*		*M. intracellulare* subsp. *intracellulare*
96	*M. intracellulare*	*M. intracellulare*		*M. intracellulare* subsp. *intracellulare*
97	*M. avium*	*M. avium*		*M. avium* subsp. *hominissuis*
98	*M. avium*	*M. avium*		*M. avium* subsp. *hominissuis*
99		*M.chelonae*		*M. chelonae*

100	*M. avium*	*M. avium*		*M. avium* subsp. *hominissuis*
101	*M. avium*	*M. avium*		*M. avium* subsp. *hominissuis*
102		*M. abscessus*		*M. abscessus* subsp. *abscessus*
103		*M. abscessus*	*M. masilliense*	*M. abscessus* subsp. *massiliense*
104	*M. avium*	*M. avium*		*M. avium* subsp. *hominissuis*
105	*M. avium*	*M. avium*		*M. avium* subsp. *hominissuis*
106		*M. peregrinum*		*M. peregrinum*
107	*M. intracellulare*	*M. intracellulare*		*M. intracellulare* subsp. *intracellulare*
108	*M. avium*	*M. avium*		*M. avium* subsp. *hominissuis*
109	*M. avium*	*M. avium*		*M. avium* subsp. *hominissuis*
110	*M. intracellulare*	*M. intracellulare*		*M. intracellulare* subsp. *intracellulare*
111	*M. avium*	*M. avium*		*M. avium* subsp. *hominissuis*
112	*M. avium*	*M. avium*		*M. avium* subsp. *hominissuis*
113	*M. avium*	*M. avium*		*M. avium* subsp. *hominissuis*
114	*M. avium*	*M. avium*		*M. avium* subsp. *hominissuis*
115	*M. avium*	*M. avium*		*M. avium* subsp. *hominissuis*
116	*M. avium*	*M. avium*		*M. avium* subsp. *hominissuis*

TRC, transcription-reverse transcription concerted reaction; MALDI-TOF MS, Matrix-assisted laser desorption ionization-time of flight mass spectrometry; PCR, polymerase chain reaction; *M. avium*, *mycobacterium avium*; subsp, subspecies.

Table S2. Data yields of MGIT sequences.

No.	BioSample Accession	DRA Accession	Sequencing data Total length (bp)	Minimum length (bp)	Mean length (bp)	Maximum length (bp)
1	SAMD00439156	DRX326587	1379445652	115	3877.6	17009
2	SAMD00439157	DRX326588	1183838471	136	3851.3	15889
3	SAMD00439158	DRX326589	1200498471	126	4238.6	15087
4	SAMD00439159	DRX326590	1503371099	106	3818.3	14976
5	SAMD00439160	DRX326591	1299444581	116	3375.8	17084
6	SAMD00439161	DRX326592	992770021	132	3711.4	16875
7	SAMD00439162	DRX326593	1317896803	133	3669.5	17632
8	SAMD00439163	DRX326594	956354734	107	3650.6	14971
9	SAMD00439164	DRX326595	800077167	135	3845.4	14482
10	SAMD00439165	DRX326596	1323810361	129	4059.8	19939
11	SAMD00439166	DRX326597	1066823604	134	3805.5	16300
12	SAMD00439167	DRX326598	1239724983	110	3716.3	19978
13	SAMD00439168	DRX326599	229480759	134	3372.3	13813
14	SAMD00439169	DRX326600	928125280	131	3437.7	15554
15	SAMD00439170	DRX326601	1047031984	137	3800.6	16265
16	SAMD00439171	DRX326602	1167334664	96	3769.9	14944
17	SAMD00439172	DRX326603	1148472749	129	3821.7	17077
18	SAMD00439173	DRX326604	976344608	97	4517.9	17943
19	SAMD00439174	DRX326605	1587223096	114	4643.2	15271
20	SAMD00439175	DRX326606	319526749	123	3820.8	18184
21	SAMD00439176	DRX326607	757293399	140	3827	21019

22	SAMD00439177	DRX326608	938978688	120	3779.5	17809
23	SAMD00439178	DRX326609	811807286	120	3619	16627
24	SAMD00439179	DRX326610	957081431	136	3697.2	15663
25	SAMD00439180	DRX326611	376356736	130	3694.4	14371
26	SAMD00439181	DRX326612	261965123	116	3257	18781
27	SAMD00439182	DRX326613	271625377	150	3430.5	18355
28	SAMD00439183	DRX326614	901895250	138	3778.4	14209
29	SAMD00439184	DRX326615	1469055292	132	3888.9	15846
30	SAMD00439185	DRX326616	1473239748	135	4279.9	19117
31	SAMD00439186	DRX326617	1248405874	95	4003.4	20143
32	SAMD00439187	DRX326618	1023938864	121	4818.3	16992
33	SAMD00439188	DRX326619	1082227265	119	3844	15341
34	SAMD00439189	DRX326620	1718569071	119	3691.8	14216
35	SAMD00439190	DRX326621	1983725397	110	3654.7	17816
36	SAMD00439191	DRX326622	1450617935	120	4205.4	16049
37	SAMD00439192	DRX326623	1255392074	111	4504.3	17979
38	SAMD00439193	DRX326624	1351768155	119	4102.1	18415
39	SAMD00439194	DRX326625	1561113896	120	4253.1	14844
40	SAMD00439195	DRX326626	87883840	125	3962.5	13634
41	SAMD00439196	DRX326627	773680753	113	3523.4	15631
42	SAMD00439197	DRX326628	294857625	123	3416.7	19571
43	SAMD00439198	DRX326629	1131894374	102	4052	15219
44	SAMD00439199	DRX326630	2826623143	112	3671.6	16927

45	SAMD00439200	DRX326631	1761544879	102	3971.8	15604
46	SAMD00439201	DRX326632	1260932688	109	4055.9	14056
47	SAMD00439202	DRX326633	1232212083	112	4115.7	18065
48	SAMD00439203	DRX326634	1084736367	88	4322.7	15485
49	SAMD00439204	DRX326635	269137843	136	3646.3	16168
50	SAMD00439205	DRX326636	1851744221	112	3751.7	23485
51	SAMD00439206	DRX326637	1830126149	110	3913.8	16957
52	SAMD00439207	DRX326638	1848757978	105	3787.1	14998
53	SAMD00439208	DRX326639	2115250083	106	4010.7	20031
54	SAMD00514954	DRX381021	1104379408	50	4274.4	103586
55	SAMD00514955	DRX381022	1723567831	27	3484.6	99991
56	SAMD00514956	DRX381023	1151099911	38	4081.6	124817
57	SAMD00514957	DRX381024	1321094465	36	3848	64398
58	SAMD00514958	DRX381025	1104261416	54	4447	111676
59	SAMD00514959	DRX381026	1371315996	39	3481.3	15435
60	SAMD00514960	DRX381027	1049420519	44	4229.1	17370
61	SAMD00514961	DRX381028	825265882	50	4349	63574
62	SAMD00514962	DRX381029	1117995547	61	4600.2	111233
63	SAMD00514963	DRX381030	1215273788	55	3566.5	26188
64	SAMD00514964	DRX381031	1155317487	27	4115.2	18215
65	SAMD00514965	DRX381032	1254515168	26	3974.3	15782
66	SAMD00514966	DRX381033	1150374049	53	4115	15006
67	SAMD00514967	DRX381034	1273822158	40	3835.9	42233

68	SAMD00514968	DRX381035	1820201153	42	3971.3	20289
69	SAMD00514969	DRX381036	1354231916	30	4284.8	77356
70	SAMD00514970	DRX381037	1051530543	33	3987.5	16364
71	SAMD00514971	DRX381038	896989137	46	4501.1	17212
72	SAMD00514972	DRX381039	1609732788	19	3446.5	15004
73	SAMD00514973	DRX381040	1378073827	24	4168.4	194377
74	SAMD00514974	DRX381041	1543506009	51	3899.2	84467
75	SAMD00514975	DRX381042	1840922128	22	3853.9	16068
76	SAMD00514976	DRX381043	2042614145	47	3411.6	17845
77	SAMD00514977	DRX381044	1072291109	26	4142.2	15809
78	SAMD00514978	DRX381045	1786089159	49	3901.7	19893
79	SAMD00514979	DRX381046	1702386294	36	3691.3	17602
80	SAMD00514980	DRX381047	1514872882	43	3978.7	17488
81	SAMD00514981	DRX381048	1531081203	34	3996.9	30558
82	SAMD00514982	DRX381049	1644504644	26	3747.2	41424
83	SAMD00514983	DRX381050	1585904785	45	4003.1	16475
84	SAMD00514984	DRX381051	1465583289	35	3833.7	15660
85	SAMD00514985	DRX381052	1631414667	57	4055.1	31083
86	SAMD00514986	DRX381053	1217730069	44	3940.8	18461
87	SAMD00514987	DRX381054	1840812853	45	4027.8	15711
88	SAMD00514988	DRX381055	1105482093	35	3935.9	17229
89	SAMD00514989	DRX381056	1152559056	50	3776.7	17583
90	SAMD00514990	DRX381057	1541314457	41	3651	115285

91	SAMD00514991	DRX381058	1477392042	47	4460.7	40395
92	SAMD00514992	DRX381059	1251458910	50	3465.2	14134
93	SAMD00514993	DRX381060	1295112694	51	3945	65857
94	SAMD00514994	DRX381061	1194026247	58	3697.3	16593
95	SAMD00514995	DRX381062	1063396978	44	4100.1	16556
96	SAMD00514996	DRX381063	1319331996	52	3796.6	60204
97	SAMD00514997	DRX381064	1424891902	51	3531.5	14446
98	SAMD00514998	DRX381065	1100193455	27	3895.4	18247
99	SAMD00514999	DRX381066	1413163085	52	3357.6	16771
100	SAMD00515000	DRX381067	1322196906	46	3909.8	15731
101	SAMD00515001	DRX381068	1202968051	63	4168.6	17004
102	SAMD00515002	DRX381069	1578220472	32	4264.5	18113
103	SAMD00515003	DRX381070	1565160366	22	4095.2	15813
104	SAMD00515004	DRX381071	1799516742	45	3586.4	14629
105	SAMD00515005	DRX381072	927900876	39	3911.5	19034
106	SAMD00515006	DRX381073	1873528222	37	3614.9	16167
107	SAMD00515007	DRX381074	1421267857	16	4113.2	17618
108	SAMD00515008	DRX381075	1548612174	41	3872.9	15372
109	SAMD00515009	DRX381076	1362896054	34	3741.5	20447
110	SAMD00515010	DRX381077	1427524906	7	4064.3	16982
111	SAMD00515011	DRX381078	1392223394	58	3801.8	15663
112	SAMD00515012	DRX381079	1233353980	45	4157.4	16881
113	SAMD00515013	DRX381080	1479054437	45	4352.3	17379

114	SAMD00515014	DRX381081	1728592992	21	4113.6	17965
115	SAMD00515015	DRX381082	1766100198	49	4148.3	16118
116	SAMD00515016	DRX381083	2096124177	38	3477.6	14732

Table S3. Full analysis results of *MLST verse*

Sample	Species	Strain	Score
**#1**	***Mycobacterium avium* subsp. *hominissuis***	**Tone-15**	**0.60**
#1	*Mycobacterium avium* subsp. *avium*	DT 78	0.33
#1	*Mycobacterium timonense*	CCUG 56329	0.27
**#2**	***Mycobacterium intracellulare* subsp. *intracellulare***	ATCC 13950	**0.41**
#2	*Mycobacterium avium* subsp. *avium*	2285 (S)	0.26
#2	*Mycobacterium intracellulare* subsp. *chimaera*	FLAC0067	0.24
**#3**	***Mycobacterium avium* subsp. *hominissuis***	**IH-145**	**0.47**
#3	*Mycobacterium timonense*	CCUG 56329	0.26
#3	*Mycobacterium avium* subsp. *avium*	11-4751	0.24
**#4**	***Mycobacterium intracellulare* subsp. *intracellulare***	**MIN_061107_1834**	**0.66**
#4	*Mycobacterium avium* subsp. *avium*	2285 (S)	0.44
#4	*Mycobacterium intracellulare* subsp. *chimaera*	FLAC0067	0.25
**#5**	***Mycobacterium avium* subsp. *hominissuis***	**OCU404**	**0.48**
#5	*Mycobacterium avium* subsp. *avium*	DJO-44271	0.28
#5	*Mycobacterium timonense*	CCUG 56329	0.26
**#6**	***Mycobacterium avium* subsp. *hominissuis***	**IH-945**	**0.43**
#6	*Mycobacterium avium* subsp. *avium*	DJO-44271	0.21
#6	*Mycobacterium timonense*	CCUG 56329	0.21
**#7**	***Mycobacterium intracellulare* subsp. *intracellulare***	**852002-53206_SCH5915646**	**0.42**
#7	*Mycobacterium avium* subsp. *avium*	2285 (S)	0.24
#7	*Mycobacterium intracellulare* subsp. *chimaera*	FLAC0067	0.19
**#8**	***Mycobacterium intracellulare* subsp. *chimaera***	**CDC 2015-22-71**	**0.50**
#8	*Mycobacterium intracellulare* subsp. *intracellulare*	MIN_052511_1280	0.49
#8	*Mycobacterium avium* subsp. *avium*	2285 (S)	0.17
**#9**	***Mycobacterium avium* subsp. *hominissuis***	**IH-801**	**0.47**
#9	*Mycobacterium avium* subsp. *avium*	11-4751	0.23
#9	*Mycobacterium avium* subsp. *paratuberculosis*	FDAARGOS_305	0.20
**#10**	***Mycobacterium intracellulare* subsp. *chimaera***	**FLAC0067**	**0.74**
#10	*Mycobacterium intracellulare* subsp. *intracellulare*	FLAC0181	0.29
#10	*Mycobacterium avium* subsp. *avium*	2285 (S)	0.24
**#11**	***Mycobacterium avium* subsp. *hominissuis***	**MK20-5**	**0.49**
#11	*Mycobacterium timonense*	CCUG 56329	0.32
#11	*Mycobacterium avium* subsp. *avium*	DJO-44271	0.29
**#12**	***Mycobacterium intracellulare* subsp. *intracellulare***	**M.i.198**	**0.62**
#12	*Mycobacterium avium* subsp. *avium*	2285 (S)	0.31
#12	*Mycobacterium intracellulare* subsp. *chimaera*	SJ42	0.20
**#13**	***Mycobacterium avium* subsp. *hominissuis***	**NN-127**	**0.55**

#13	*Mycobacterium avium* subsp. *avium*	2285 (R)	0.52
#13	*Mycobacterium timonense*	CCUG 56329	0.44
**#14**	***Mycobacterium intracellulare* subsp. *intracellulare***	ATCC 13950	**0.45**
#14	*Mycobacterium avium* subsp. *avium*	2285 (S)	0.30
#14	*Mycobacterium intracellulare* subsp. *chimaera*	FLAC0070	0.27
**#15**	***Mycobacterium intracellulare* subsp. *intracellulare***	FLAC0181	0.41
#15	*Mycobacterium intracellulare* subsp. *chimaera*	FLAC0067	0.31
#15	*Mycobacterium avium* subsp. *avium*	2285 (S)	0.29
**#16**	***Mycobacterium avium* subsp. *hominissuis***	**IH-945**	**0.47**
#16	*Mycobacterium intracellulare* subsp. *chimaera*	FLAC0067	0.38
#16	*Mycobacterium avium* subsp. *avium*	DJO-44271	0.32
**#17**	***Mycobacterium kansasii***	**11-3469**	**0.77**
**#18**	***Mycobacterium lentiflavum***	**CSUR P1491**	**0.82**
**#19**	***Mycobacterium abscessus* subsp. *massiliense***	**471**	**0.97**
#19	*Mycobacterium abscessus* subsp. *bolletii*		0.65
#19	*Mycobacterium abscessus* subsp. *abscessus*	DJO-44274	0.65
**#20**	***Mycobacterium intracellulare* subsp. *intracellulare***	**MIN_061107_1834**	**0.51**
#20	*Mycobacterium avium* subsp. *avium*	2285 (S)	0.47
#20	*Mycobacterium intracellulare* subsp. *chimaera*	JCM_14737	0.18
**#21**	***Mycobacterium avium* subsp. *hominissuis***	**Tone-12**	**0.56**
#21	*Mycobacterium timonense*	CCUG 56329	0.28
#21	*Mycobacterium avium* subsp. *avium*	DJO-44271	0.28
**#22**	***Mycobacterium avium* subsp. *hominissuis***	**IH-687**	**0.54**
#22	*Mycobacterium timonense*	CCUG 56329	0.31
#22	*Mycobacterium avium* subsp. *avium*	2285 (R)	0.31
**#23**	***Mycobacterium avium* subsp. *hominissuis***	**HF-3**	**0.42**
#23	*Mycobacterium avium* subsp. *avium*	DT 78	0.27
#23	*Mycobacterium timonense*	CCUG 56329	0.25
**#24**	***Mycobacterium avium* subsp. *hominissuis***	**OCU901s_S2_2s**	**0.49**
#24	*Mycobacterium avium* subsp. *avium*	DT 78	0.23
#24	*Mycobacterium timonense*	CCUG 56329	0.20
**#25**	***Mycobacterium avium* subsp. *hominissuis***	**IH-788**	**0.44**
#25	*Mycobacterium avium* subsp. *avium*	11-4751	0.31
#25	*Mycobacterium timonense*	CCUG 56329	0.27
**#26**	***Mycobacterium avium* subsp. *hominissuis***	**CAM57**	**0.47**
#26	*Mycobacterium avium* subsp. *avium*	DJO-44271	0.34
#26	*Mycobacterium timonense*	CCUG 56329	0.29
**#27**	***Mycobacterium avium* subsp. *hominissuis***	**TR-M-4**	**0.48**
#27	*Mycobacterium avium* subsp. *avium*	DT 78	0.25

#27	*Mycobacterium avium* subsp. *paratuberculosis*	11-1786	0.20
**#28**	***Mycobacterium avium* subsp. *hominissuis***	**IH-149**	**0.48**
#28	*Mycobacterium avium* subsp. *avium*	DJO-44271	0.28
#28	*Mycobacterium timonense*	CCUG 56329	0.27
**#29**	***Mycobacterium intracellulare* subsp. *intracellulare***	**852002-53206_SCH5915646**	**0.33**
#29	*Mycobacterium avium* subsp. *avium*	2285 (S)	0.29
#29	*Mycobacterium intracellulare* subsp. *chimaera*	FLAC0067	0.25
**#30**	***Mycobacterium intracellulare* subsp. *intracellulare***	**FLAC0162**	**0.39**
#30	*Mycobacterium intracellulare* subsp. *chimaera*	FLAC0067	0.34
#30	*Mycobacterium avium* subsp. *avium*	2285 (S)	0.31
**#31**	***Mycobacterium intracellulare* subsp. *intracellulare***	**MIN_061107_1834**	**0.61**
#31	*Mycobacterium avium* subsp. *avium*	2285 (S)	0.45
#31	*Mycobacterium intracellulare* subsp. *chimaera*	FLAC0067	0.21
**#32**	***Mycobacterium abscessus* subsp. *abscessus***	**1027**	**0.98**
#32	*Mycobacterium abscessus* subsp. *bolletii*	103	0.77
#32	*Mycobacterium fortuitum*	Z58	0.70
**#33**	***Mycobacterium abscessus* subsp. *massiliense***	**922**	**0.98**
#33	*Mycobacterium abscessus* subsp. *bolletii*		0.70
#33	*Mycobacterium abscessus* subsp. *abscessus*	199	0.40
**#34**	***Mycobacterium avium* subsp. *hominissuis***	**Tone-12**	**0.58**
#34	*Mycobacterium avium* subsp. *avium*	11-4751	0.26
#34	*Mycobacterium avium* subsp. *silvaticum*	ATCC 49884	0.21
**#35**	***Mycobacterium avium* subsp. *hominissuis***	**OCU466**	**0.47**
#35	*Mycobacterium timonense*	CCUG 56329	0.32
#35	*Mycobacterium avium* subsp. *avium*	11-4751	0.27
**#36**	***Mycobacterium avium* subsp. *hominissuis***	**CAM57**	**0.43**
#36	*Mycobacterium avium* subsp. *avium*	11-4751	0.25
#36	*Mycobacterium timonense*	CCUG 56329	0.20
**#37**	***Mycobacterium avium* subsp. *hominissuis***	**OCU901s_S2_2s**	**0.52**
#37	*Mycobacterium avium* subsp. *avium*	DJO-44271	0.25
#37	*Mycobacterium timonense*	CCUG 56329	0.22
**#38**	***Mycobacterium avium* subsp. *hominissuis***	**IH-217**	**0.60**
#38	*Mycobacterium avium* subsp. *avium*	11-4751	0.26
#38	*Mycobacterium avium* subsp. *silvaticum*	ATCC 49884	0.23
**#39**	***Mycobacterium intracellulare* subsp. *intracellulare***	**MIN_061107_1834**	**0.56**
#39	*Mycobacterium avium* subsp. *avium*	2285 (S)	0.45
#39	*Mycobacterium intracellulare* subsp. *chimaera*	SJ42	0.21
**#40**	***Mycobacterium avium* subsp. *hominissuis***	**TH135**	**0.40**
#40	*Mycobacterium timonense*	CCUG 56329	0.20

#40	*Mycobacterium avium* subsp. *avium*	11-4751	0.20
**#41**	***Mycobacterium avium* subsp. *hominissuis***	**TR-M-4**	**0.66**
#41	*Mycobacterium avium* subsp. *avium*	11-4751	0.23
#41	*Mycobacterium timonense*	CCUG 56329	0.21
**#42**	***Mycobacterium avium* subsp. *hominissuis***	**IH-550**	**0.63**
#42	*Mycobacterium avium* subsp. *avium*	11-4751	0.40
#42	*Mycobacterium timonense*	CCUG 56329	0.40
**#43**	***Mycobacterium paragordonae***		**0.63**
#43	*Mycobacterium gordonae*	1275229.4	0.54
**#44**	***Mycobacterium abscessus* subsp. *massiliense***	**1140**	**0.84**
#44	*Mycobacterium abscessus* subsp. *bolletii*	M18	0.68
#44	*Mycobacterium abscessus* subsp. *abscessus*	DJO-44274	0.67
**#45**	***Mycobacterium avium* subsp. *hominissuis***	**IH-145**	**0.48**
#45	*Mycobacterium avium* subsp. *avium*	11-4751	0.21
#45	*Mycobacterium avium* subsp. *paratuberculosis*	10-5975	0.21
**#46**	***Mycobacterium avium* subsp. *hominissuis***	**NN-108**	**0.58**
#46	*Mycobacterium avium* subsp. *avium*	DJO-44271	0.30
#46	*Mycobacterium timonense*	CCUG 56329	0.28
**#47**	***Mycobacterium avium* subsp. *hominissuis***	**OCU901s_S2_2s**	**0.48**
#47	*Mycobacterium avium* subsp. *avium*	11-4751	0.27
#47	*Mycobacterium avium* subsp. *silvaticum*	ATCC 49884	0.24
**#48**	***Mycobacterium avium* subsp. *hominissuis***	**Tone-15**	**0.41**
#48	*Mycobacterium avium* subsp. *avium*	11-4751	0.27
#48	*Mycobacterium timonense*	CCUG 56329	0.22
**#49**	***Mycobacterium avium* subsp. *hominissuis***	**Tone-13**	**0.60**
#49	*Mycobacterium avium* subsp. *avium*	11-4751	0.23
#49	*Mycobacterium timonense*	CCUG 56329	0.22
**#50**	***Mycobacterium intracellulare* subsp. *intracellulare***	**MIN_061107_1834**	**0.50**
#50	*Mycobacterium avium* subsp. *avium*	2285 (S)	0.38
#50	*Mycobacterium intracellulare* subsp. *chimaera*	FLAC0067	0.23
**#51**	***Mycobacterium intracellulare* subsp. *intracellulare***	**ATCC 13950**	**0.45**
#51	*Mycobacterium avium* subsp. *avium*	2285 (S)	0.38
#51	*Mycobacterium intracellulare* subsp. *chimaera*	JCM_14737	0.21
**#52**	***Mycobacterium avium* subsp. *hominissuis***	**Tone-12**	**0.54**
#52	*Mycobacterium avium* subsp. *avium*	DJO-44271	0.28
#52	*Mycobacterium timonense*	CCUG 56329	0.27
**#53**	***Mycobacterium gordonae***	**CTRI 14-8773**	**0.81**
**#54**	***Mycobacterium avium* subsp. *hominissuis***	**TR-M-4**	**0.68**
#54	*Mycobacterium avium* subsp. *avium*	2285 (R)	0.44

#54	*Mycobacterium avium* subsp. *paratuberculosis*	10-4404	0.43
**#55**	***Mycobacterium avium* subsp. *hominissuis***	**IH-687**	**0.63**
#55	*Mycobacterium timonense*	CCUG 56329	0.48
#55	*Mycobacterium avium* subsp. *avium*	DJO-44271	0.46
**#56**	***Mycobacterium avium* subsp. *hominissuis***	**OCU901s_S2_2s**	**0.68**
#56	*Mycobacterium avium* subsp. *avium*	11-4751	0.35
#56	*Mycobacterium timonense*	CCUG 56329	0.34
**#57**	***Mycobacterium avium* subsp. *hominissuis***	**OCU462**	**0.55**
#57	*Mycobacterium timonense*	CCUG 56329	0.45
#57	*Mycobacterium avium* subsp. *avium*	2285 (R)	0.42
**#58**	***Mycobacterium avium* subsp. *hominissuis***	**IH-217**	**0.66**
#58	*Mycobacterium avium* subsp. *avium*	2285 (R)	0.47
#58	*Mycobacterium timonense*	CCUG 56329	0.45
**#59**	***Mycobacterium intracellulare* subsp. *intracellulare***	**ATCC 13950**	**0.72**
#59	*Mycobacterium avium* subsp. *avium*	2285 (S)	0.62
#59	*Mycobacterium intracellulare* subsp. *chimaera*	FLAC0067	0.55
**#60**	***Mycobacterium avium* subsp. *hominissuis***	**OCU404**	**0.58**
#60	*Mycobacterium timonense*	CCUG 56329	0.47
#60	*Mycobacterium avium* subsp. *avium*	11-4751	0.44
**#61**	***Mycobacterium avium* subsp. *hominissuis***	**IH-065**	**0.63**
#61	*Mycobacterium timonense*	CCUG 56329	0.51
#61	*Mycobacterium avium* subsp. *avium*	DJO-44271	0.47
**#62**	***Mycobacterium lentiflavum***	**CSUR P1491**	**0.92**
#62	*Mycobacterium triplex*	DSM 44626	0.15
#62	*Mycobacterium stomatepiae*		0.15
**#63**	***Mycobacterium avium* subsp. *hominissuis***	**Tone-1**	**0.57**
#63	*Mycobacterium avium* subsp. *avium*	ATCC 25291	0.40
#63	*Mycobacterium timonense*	CCUG 56329	0.39
**#64**	***Mycobacterium avium* subsp. *hominissuis***	**OCU466**	**0.61**
#64	*Mycobacterium timonense*	CCUG 56329	0.52
#64	*Mycobacterium avium* subsp. *avium*	11-4751	0.50
**#65**	***Mycobacterium avium* subsp. *hominissuis***	**DH-6**	**0.73**
#65	*Mycobacterium avium* subsp. *avium*	DJO-44271	0.50
#65	*Mycobacterium timonense*	CCUG 56329	0.49
**#66**	***Mycobacterium avium* subsp. *hominissuis***	**OCU901s_S2_2s**	**0.69**
#66	*Mycobacterium avium* subsp. *avium*	11-4751	0.37
#66	*Mycobacterium timonense*	CCUG 56329	0.36
**#67**	***Mycobacterium avium* subsp. *hominissuis***	**OCU901s_S2_2s**	**0.62**
#67	*Mycobacterium timonense*	CCUG 56329	0.47

#67	*Mycobacterium avium* subsp. *avium*	2285 (R)	0.46
**#68**	***Mycobacterium avium* subsp. *hominissuis***	**DH-5**	**0.60**
#68	*Mycobacterium avium* subsp. *avium*	DJO-44271	0.42
#68	*Mycobacterium timonense*	CCUG 56329	0.42
**#69**	***Mycobacterium porcinum***	**IP141460001**	**0.94**
#69	*Mycobacterium boenickei*		0.47
#69	*Mycobacterium neworleansense*	type strain: ATCC 49404	0.22
**#70**	***Mycobacterium avium* subsp. *hominissuis***	**IH-065**	**0.54**
#70	*Mycobacterium timonense*	CCUG 56329	0.46
#70	*Mycobacterium avium* subsp. *avium*	2285 (R)	0.43
**#71**	***Mycobacterium szulgai***	**DSM 44166**	**0.88**
#71	*Mycobacterium angelicum*	DSM 45057	0.38
**#72**	***Mycobacterium abscessus* subsp. *abscessus***	**522**	**0.98**
#72	*Mycobacterium fortuitum*	Z58	0.87
#72	*Mycobacterium abscessus* subsp. *bolletii*	103	0.80
**#73**	***Mycobacterium fortuitum***	**E1336**	**0.91**
**#73**	***Mycobacterium fortuitum* subsp. *fortuitum***	**ATCC 6841**	**0.86**
#73	*Mycobacterium fortuitum* subsp. *acetamidolyticum*	JCM6368	0.80
**#74**	***Mycobacterium peregrinum***	**DSM 43271**	**0.94**
#74	*Mycobacterium septicum*	type strain: DSM 44393	0.23
#74	*Mycobacterium neworleansense*	type strain: ATCC 49404	0.20
**#75**	***Mycobacterium avium* subsp. *hominissuis***	**OCU404**	**0.67**
#75	*Mycobacterium avium* subsp. *avium*	DJO-44271	0.47
#75	*Mycobacterium timonense*	CCUG 56329	0.44
**#76**	***Mycobacterium intracellulare* subsp. *intracellulare***	**MOTT-02**	**0.79**
#76	*Mycobacterium avium* subsp. *avium*	2285 (S)	0.65
#76	*Mycobacterium intracellulare* subsp. *chimaera*	FLAC0067	0.55
**#77**	***Mycobacterium avium* subsp. *hominissuis***	**OCU901s_S2_2s**	**0.63**
#77	*Mycobacterium timonense*	CCUG 56329	0.45
#77	*Mycobacterium avium* subsp. *avium*	2285 (R)	0.44
**#78**	***Mycobacterium intracellulare* subsp. *intracellulare***	**MIN_061107_1834**	**0.64**
#78	*Mycobacterium avium* subsp. *avium*	2285 (S)	0.59
#78	*Mycobacterium intracellulare* subsp. *chimaera*	FLAC0067	0.45
**#79**	***Mycobacterium avium* subsp. *hominissuis***	**TR-M-4**	**0.65**
#79	*Mycobacterium avium* subsp. *avium*	2285 (R)	0.37
#79	*Mycobacterium timonense*	CCUG 56329	0.36
**#80**	***Mycobacterium intracellulare* subsp. *intracellulare***	**ATCC 13950**	**0.65**
#80	*Mycobacterium avium* subsp. *avium*	2285 (S)	0.60
#80	*Mycobacterium intracellulare* subsp. *chimaera*	FLAC0067	0.51

**#81**	***Mycobacterium avium* subsp. *hominissuis***	**IH-145**	**0.66**
#81	*Mycobacterium avium* subsp. *paratuberculosis*	10-4404	0.39
#81	*Mycobacterium timonense*	CCUG 56329	0.39
**#82**	***Mycobacterium avium* subsp. *hominissuis***	**TR-M-3**	**0.71**
#82	*Mycobacterium avium* subsp. *avium*	DJO-44271	0.41
#82	*Mycobacterium timonense*	CCUG 56329	0.40
**#83**	***Mycobacterium avium* subsp. *hominissuis***	**CAM57**	**0.67**
#83	*Mycobacterium avium* subsp. *avium*	DJO-44271	0.47
#83	*Mycobacterium timonense*	CCUG 56329	0.44
**#84**	***Mycobacterium intracellulare* subsp. *intracellulare***	**MIN_061107_1834**	**0.77**
#84	*Mycobacterium avium* subsp. *avium*	2285 (S)	0.66
#84	*Mycobacterium intracellulare* subsp. *chimaera*	FLAC0067	0.53
**#85**	***Mycobacterium paragordonae***		**0.75**
#85	*Mycobacterium gordonae*	1275229.4	0.68
**#86**	***Mycobacterium avium* subsp. *hominissuis***	**IH-217**	**0.68**
#86	*Mycobacterium avium* subsp. *avium*	2285 (R)	0.45
#86	*Mycobacterium timonense*	CCUG 56329	0.43
**#87**	***Mycobacterium intracellulare* subsp. *intracellulare***	**M.i.198**	**0.67**
#87	*Mycobacterium avium* subsp. *avium*	2285 (S)	0.54
#87	*Mycobacterium intracellulare* subsp. *chimaera*	FLAC0067	0.43
**#88**	***Mycobacterium avium* subsp. *hominissuis***	**DH-8-4**	**0.52**
#88	*Mycobacterium avium* subsp. *avium*	2285 (S)	0.43
#88	*Mycobacterium timonense*	CCUG 56329	0.40
**#89**	***Mycobacterium intracellulare* subsp. *intracellulare***	**ATCC 13950**	**0.74**
#89	*Mycobacterium avium* subsp. *avium*	2285 (S)	0.64
#89	*Mycobacterium intracellulare* subsp. *chimaera*	FLAC0067	0.51
**#90**	***Mycobacterium abscessus* subsp. *massiliense***	**932**	**1.00**
#90	*Mycobacterium abscessus* subsp. *bolletii*	MM1513	0.88
#90	*Mycobacterium abscessus* subsp. *abscessus*	199	0.60
**#91**	***Mycobacterium intracellulare* subsp. *intracellulare***	**FLAC0133**	**0.64**
#91	*Mycobacterium avium* subsp. *avium*	2285 (S)	0.57
#91	*Mycobacterium intracellulare* subsp. *chimaera*	FLAC0067	0.47
**#92**	***Mycobacterium avium* subsp. *hominissuis***	**CAM177**	**0.67**
#92	*Mycobacterium timonense*	CCUG 56329	0.43
#92	*Mycobacterium avium* subsp. *avium*	11-4751	0.42
**#93**	***Mycobacterium paragordonae***		**0.73**
#93	*Mycobacterium gordonae*	1275229.4	0.69
**#94**	***Mycobacterium avium* subsp. *hominissuis***	**TR-M-4**	**0.73**
#94	*Mycobacterium avium* subsp. *avium*	11-4751	0.40

#94	*Mycobacterium timonense*	CCUG 56329	0.39
**#95**	***Mycobacterium intracellulare* subsp. *intracellulare***	**FLAC0181**	**0.69**
#95	*Mycobacterium avium* subsp. *avium*	2285 (S)	0.59
#95	*Mycobacterium intracellulare* subsp. *chimaera*	FLAC0067	0.54
**#96**	***Mycobacterium intracellulare* subsp. *intracellulare***	**FLAC0162**	**0.65**
#96	*Mycobacterium avium* subsp. *avium*	2285 (S)	0.61
#96	*Mycobacterium intracellulare* subsp. *chimaera*	FLAC0067	0.45
**#97**	***Mycobacterium avium* subsp. *hominissuis***	**OCU466**	**0.56**
#97	*Mycobacterium timonense*	CCUG 56329	0.45
#97	*Mycobacterium avium* subsp. *avium*	2285 (R)	0.43
**#98**	***Mycobacterium avium* subsp. *hominissuis***	**TR-M-4**	**0.58**
#98	*Mycobacterium avium* subsp. *avium*	11-4751	0.36
#98	*Mycobacterium timonense*	CCUG 56329	0.32
**#99**	***Mycobacterium chelonae***	**24998**	**1.00**
#99	*Mycobacterium stephanolepidis*	NJB0901	0.28
**#100**	***Mycobacterium avium* subsp. *hominissuis***	**IH-550**	**0.68**
#100	*Mycobacterium avium* subsp. *avium*	DJO-44271	0.51
#100	*Mycobacterium timonense*	CCUG 56329	0.48
**#101**	***Mycobacterium avium* subsp. *hominissuis***	**TR-M-4**	**0.72**
#101	*Mycobacterium avium* subsp. *avium*	2285 (R)	0.38
#101	*Mycobacterium timonense*	CCUG 56329	0.38
**#102**	***Mycobacterium abscessus* subsp. *abscessus***	**1106**	**1.00**
#102	*Mycobacterium fortuitum*	Z58	0.90
#102	*Mycobacterium abscessus* subsp. *bolletii*	103	0.83
**#103**	***Mycobacterium abscessus* subsp. *massiliense***	**1141**	**1.00**
#103	*Mycobacterium abscessus* subsp. *bolletii*		0.82
#103	*Mycobacterium abscessus* subsp. *abscessus*	199	0.57
**#104**	***Mycobacterium avium* subsp. *hominissuis***	**Tone-16**	**0.57**
#104	*Mycobacterium avium* subsp. *avium*	DT 78	0.46
#104	*Mycobacterium avium* subsp. *paratuberculosis*	10-4404	0.44
**#105**	***Mycobacterium avium* subsp. *hominissuis***	**11**	**0.59**
#105	*Mycobacterium avium* subsp. *avium*	DT 78	0.47
#105	*Mycobacterium timonense*	CCUG 56329	0.37
**#106**	***Mycobacterium peregrinum***	**DSM 43271**	**0.90**
#106	*Mycobacterium porcinum*	IP141460001	0.19
#106	*Mycobacterium vulneris*	DSM 45247	0.19
**#107**	***Mycobacterium intracellulare* subsp. *intracellulare***	**FLAC0162**	**0.73**
#107	*Mycobacterium avium* subsp. *avium*	2285 (S)	0.61
#107	*Mycobacterium intracellulare* subsp. *chimaera*	FLAC0067	0.50

**#108**	***Mycobacterium avium* subsp. *hominissuis***	**TR-M-1**	**0.64**
#108	*Mycobacterium avium* subsp. *avium*	DJO-44271	0.46
#108	*Mycobacterium timonense*	CCUG 56329	0.45
**#109**	***Mycobacterium avium* subsp. *hominissuis***	**TR-M-1**	**0.69**
#109	*Mycobacterium timonense*	CCUG 56329	0.54
#109	*Mycobacterium avium* subsp. *avium*	DT 78	0.49
**#110**	***Mycobacterium intracellulare* subsp. *intracellulare***	**FLAC0181**	**0.74**
#110	*Mycobacterium avium* subsp. *avium*	2285 (S)	0.67
#110	*Mycobacterium intracellulare* subsp. *chimaera*	FLAC0067	0.61
**#111**	***Mycobacterium avium* subsp. *hominissuis***	**OCU404**	**0.58**
#111	*Mycobacterium avium* subsp. *avium*	2285 (R)	0.41
#111	*Mycobacterium timonense*	CCUG 56329	0.40
**#112**	***Mycobacterium avium* subsp. *hominissuis***	**IH-217**	**0.62**
#112	*Mycobacterium timonense*	CCUG 56329	0.41
#112	*Mycobacterium avium* subsp. *avium*	DJO-44271	0.39
**#113**	***Mycobacterium avium* subsp. *hominissuis***	**TR-M-4**	**0.69**
#113	*Mycobacterium avium* subsp. *avium*	DJO-44271	0.39
#113	*Mycobacterium timonense*	CCUG 56329	0.38
**#114**	***Mycobacterium avium* subsp. *hominissuis***	**OCU901s_S2_2s**	**0.56**
#114	*Mycobacterium avium* subsp. *avium*	DJO-44271	0.39
#114	*Mycobacterium timonense*	CCUG 56329	0.38
**#115**	***Mycobacterium avium* subsp. *hominissuis***	**DH-7**	**0.65**
#115	*Mycobacterium avium* subsp. *avium*	DJO-44271	0.50
#115	*Mycobacterium timonense*	CCUG 56329	0.49
**#116**	***Mycobacterium avium* subsp. *hominissuis***	**DH-7**	**0.69**
#116	*Mycobacterium avium* subsp. *avium*	DJO-44271	0.56
#116	*Mycobacterium timonense*	CCUG 56329	0.53

MLST, multi-locus sequence typing; sp, species; subsp, subspecies.

Table S4. Whole genome comparison using ANI calculation

No.	Species name	Strain	RefSeq/GenBank accession	Type strain?	RefSeq category	ANI (%)
No.53	*Mycobacterium paragordonae*	JCM 18565	GCF_010723415.1	Yes	na	88.1
	Mycobacterium gordonae	DSM 44160	GCF_002101675.1	Yes	representative genome	86.5
	Mycobacterium sp. 20KCMC460	20KCMC460	GCF_022179425.1	No	na	81.2
	Mycobacterium sp. IWGMT90018-18076	IWGMT90018	GCF_021654635.1	No	na	80.8
	Mycobacterium asiaticum	DSM 44297	GCF_000613245.1	Yes	representative genome	80.5
	Mycobacterium vicinigordonae	24	GCF_013466425.1	Yes	representative genome	79.8
	Mycobacterium sp. MAC_011194_8550	MAC_011194_8550	GCF_000523635.1	No	na	79.7
	Mycobacterium avium	CECT 7407	GCF_015708165.1	No	na	79.7
	Mycobacterium paraense	IEC26	GCF_002101815.1	Yes	representative genome	79.7
	Mycobacterium avium subsp. *avium*	FDAARGOS_1608	GCF_021183845.1	No	na	79.7
No.71	*Mycobacterium szulgai*	DSM 44166	GCF_002116635.1	Yes	representative genome	99.4
	Mycobacterium angelicum	DSM 45057	GCF_002086155.1	Yes	representative genome	93.1
	Mycobacterium simulans	FI-09026	GCF_900232995.1	Yes	representative genome	82.1
	Mycobacterium riyadhense	DSM 45176	GCF_002101845.1	Yes	na	82.0
	Mycobacterium lacus	JCM 15657	GCF_010731535.1	Yes	representative genome	81.3

	Mycobacterium shinjukuense	JCM 14233	GCF_010730055.1	Yes	representative genome	81.1
	Mycobacterium heidelbergense	JCM 14842	GCF_010730745.1	Yes	representative genome	81.0
	Mycobacterium malmoense	ATCC 29571	GCF_019645855.1	Yes	representative genome	80.9
	Mycobacterium ostraviense	FDAARGOS_1613	GCF_021183725.1	No	representative genome	80.8
	Mycobacterium sp. E3198	E3198	GCF_001667015.1	No	na	80.7
No.85	*Mycobacterium paragordonae*	JCM 18565	GCF_010723415.1	Yes	na	87.7
	Mycobacterium gordonae	DSM 44160	GCF_002101675.1	Yes	representative genome	86.0
	Mycobacterium sp. IWGMT90018-18076	IWGMT90018	GCF_021654635.1	No	na	81.8
	Mycobacterium sp. 20KCMC460	20KCMC460	GCF_022179425.1	No	na	81.6
	Mycobacterium asiaticum	DSM 44297	GCF_000613245.1	Yes	representative genome	81.3
	Mycobacterium vicinigordonae	24	GCF_013466425.1	Yes	representative genome	80.6
	Mycobacterium paraense	IEC26	GCF_002101815.1	Yes	representative genome	80.6
	Mycobacterium avium subsp. *hominissuis*	A5	GCF_000696715.1	No	representative genome	80.6
	Mycobacterium timonense	CCUG 56329	GCF_002086775.1	Yes	representative genome	80.5
	Mycobacterium avium	CECT 7407	GCF_015708165.1	No	na	80.4
No.93	*Mycobacterium paragordonae*	JCM 18565	GCF_010723415.1	Yes	na	87.5

Mycobacterium gordonae	DSM 44160	GCF_002101675.1	Yes	representative genome	86.1
Mycobacterium sp. IWGMT90018-18076	IWGMT90018	GCF_021654635.1	No	na	81.6
Mycobacterium sp. 20KCMC460	20KCMC460	GCF_022179425.1	No	na	81.5
Mycobacterium asiaticum	DSM 44297	GCF_000613245.1	Yes	representative genome	81.2
Mycobacterium vicinigordonae	24	GCF_013466425.1	Yes	representative genome	80.4
Mycobacterium avium subsp. *paratuberculosis*	MAP-Gt-9	GCF_021696875.1	No	na	80.4
Mycobacterium avium subsp. *hominissuis*	A5	GCF_000696715.1	No	representative genome	80.4
Mycobacterium sp. MAC_011194_8550	MAC_011194_8550	GCF_000523635.1	No	na	80.3
Mycobacterium timonense	CCUG 56329	GCF_002086775.1	Yes	representative genome	80.3

ANI, average nucleotide identity; sp, species; subsp, subspecies.

Table S5. Efficacy of identification results and macrolide resistance prediction by MGIT-seq in comparison of current standard identification methods and drug susceptibility tests.

	Standard clinical protocol			Identification and macrolide resistance prediction by *MLSTverse* and MinION					
No.	TRC, MALDI-TOF MS, and Multiplex PCR and chromatographic detection	Drug susceptibility test		*MLSTverse*	*rrl* position		Erm (41) position		Predicted Macrolide susceptibility
		CAM	MIC		2058	2059	state	28	
32	*M. abscessus/bolletii*	R	>32	*M. abscessus* subsp. *abscessus*	A	A	Complete	T	R
72	*M. abscessus/bolletii*	R	>32	*M. abscessus* subsp. *abscessus*	A	A	Complete	T	R
102	*M. abscessus/bolletii*	R	>32	*M. abscessus* subsp. *abscessus*	A	A	Complete	T	R
19	*M. massiliense*	S	0.125	*M. abscessus* subsp. *massiliense*	A	A	Truncated	T	S
33	*M. massiliense*	S	0.25	*M. abscessus* subsp. *massiliense*	A	A	Truncated	T	S
44	*M. massiliense*	S	<=0.06	*M. abscessus* subsp. *massiliense*	A	A	Truncated	T	S
90	*M. massiliense*	S	0.25	*M. abscessus* subsp. *massiliense*	A	A	Truncated	T	S
103	*M. massiliense*	S	<=0.06	*M. abscessus* subsp. *massiliense*	A	A	Truncated	T	S
1	*M. avium*	S	0.5	*M. avium* subsp. *hominissuis*	A	A	None		S
3	*M. avium*	S	0.25	*M. avium* subsp. *hominissuis*	A	A	None		S
5	*M. avium*	R	>32	*M. avium* subsp. *hominissuis*	T	A	None		R
6	*M. avium*	S	0.25	*M. avium* subsp. *hominissuis*	A	A	None		S
9	*M. avium*	R	>32	*M. avium* subsp. *hominissuis*	A	C	None		R
11	*M. avium*	S	0.25	*M. avium* subsp. *hominissuis*	A	A	None		S
13	*M. avium*	R	>32	*M. avium* subsp. *hominissuis*	C	A	None		R
16	*M. avium*	R	>32	*M. avium* subsp. *hominissuis*	G	A	None		R

21	*M. avium*	S	0.5	*M. avium* subsp. *hominissuis*	A	A	None	S
22	*M. avium*	R	>32	*M. avium* subsp. *hominissuis*	A	G	None	R
23	*M. avium*	S	0.5	*M. avium* subsp. *hominissuis*	A	A	None	S
24	*M. avium*	S	0.25	*M. avium* subsp. *hominissuis*	A	A	None	S
25	*M. avium*	R	>32	*M. avium* subsp. *hominissuis*	A	A	None	S
26	*M. avium*	R	>32	*M. avium* subsp. *hominissuis*	C	A	None	R
27	*M. avium*	S	0.5	*M. avium* subsp. *hominissuis*	A	A	None	S
28	*M. avium*	S	0.5	*M. avium* subsp. *hominissuis*	A	A	None	S
34	*M. avium*	S	0.25	*M. avium* subsp. *hominissuis*	A	A	None	S
35	*M. avium*	S	4	*M. avium* subsp. *hominissuis*	A	A	None	S
36	*M. avium*	S	1	*M. avium* subsp. *hominissuis*	A	A	None	S
37	*M. avium*	R	>32	*M. avium* subsp. *hominissuis*	A	C	None	R
38	*M. avium*	S	1	*M. avium* subsp. *hominissuis*	A	A	None	S
40	*M. avium*	S	4	*M. avium* subsp. *hominissuis*	T	A	None	R
41	*M. avium*	S	0.25	*M. avium* subsp. *hominissuis*	A	A	None	S
42	*M. avium*	S	1	*M. avium* subsp. *hominissuis*	A	A	None	S
45	*M. avium*	S	0.125	*M. avium* subsp. *hominissuis*	A	A	None	S
46	*M. avium*	S	2	*M. avium* subsp. *hominissuis*	A	A	None	S
47	*M. avium*	S	0.5	*M. avium* subsp. *hominissuis*	A	A	None	S
48	*M. avium*	S	0.25	*M. avium* subsp. *hominissuis*	A	A	None	S
49	*M. avium*	S	1	*M. avium* subsp. *hominissuis*	A	A	None	S
52	*M. avium*	S	0.25	*M. avium* subsp. *hominissuis*	A	A	None	S
54	*M. avium*	S	4	*M. avium* subsp. *hominissuis*	A	A	None	S

55	*M. avium*	S	0.25	*M. avium* subsp. *hominissuis*	A	A	None	S
56	*M. avium*	S	0.5	*M. avium* subsp. *hominissuis*	A	A	None	S
57	*M. avium*	S	2	*M. avium* subsp. *hominissuis*	A	G	None	R
58	*M. avium*	S	0.125	*M. avium* subsp. *hominissuis*	A	A	None	S
60	*M. avium*	S	0.5	*M. avium* subsp. *hominissuis*	A	A	None	S
61	*M. avium*	S	0.25	*M. avium* subsp. *hominissuis*	A	A	None	S
63	*M. avium*	S	2	*M. avium* subsp. *hominissuis*	A	A	None	S
64	*M. avium*	S	0.25	*M. avium* subsp. *hominissuis*	A	A	None	S
65	*M. avium*	S	0.5	*M. avium* subsp. *hominissuis*	A	A	None	S
66	*M. avium*	S	0.5	*M. avium* subsp. *hominissuis*	A	A	None	S
67	*M. avium*	S	0.25	*M. avium* subsp. *hominissuis*	A	A	None	S
68	*M. avium*	S	0.5	*M. avium* subsp. *hominissuis*	A	A	None	S
70	*M. avium*	S	0.25	*M. avium* subsp. *hominissuis*	A	A	None	S
75	*M. avium*	S	0.25	*M. avium* subsp. *hominissuis*	A	A	None	S
77	*M. avium*	R	>32	*M. avium* subsp. *hominissuis*	A	C	None	R
79	*M. avium*	R	>32	*M. avium* subsp. *hominissuis*	G	A	None	R
81	*M. avium*	R	>32	*M. avium* subsp. *hominissuis*	C	A	None	R
82	*M. avium*	S	0.5	*M. avium* subsp. *hominissuis*	A	A	None	S
83	*M. avium*	R	>32	*M. avium* subsp. *hominissuis*	G	A	None	R
86	*M. avium*	S	2	*M. avium* subsp. *hominissuis*	A	A	None	S
88	*M. avium*	S	2	*M. avium* subsp. *hominissuis*	A	A	None	S
92	*M. avium*	S	0.125	*M. avium* subsp. *hominissuis*	A	A	None	S
94	*M. avium*	S	2	*M. avium* subsp. *hominissuis*	A	A	None	S

97	*M. avium*	S	0.5	*M. avium* subsp. *hominissuis*	A	A	None	S
98	*M. avium*	S	0.5	*M. avium* subsp. *hominissuis*	A	A	None	S
100	*M. avium*	S	2	*M. avium* subsp. *hominissuis*	A	A	None	S
101	*M. avium*	S	0.25	*M. avium* subsp. *hominissuis*	A	A	None	S
104	*M. avium*	S	0.06	*M. avium* subsp. *hominissuis*	A	A	None	S
105	*M. avium*	R	>32	*M. avium* subsp. *hominissuis*	C	A	None	R
108	*M. avium*	S	1	*M. avium* subsp. *hominissuis*	A	A	None	S
109	*M. avium*	S	1	*M. avium* subsp. *hominissuis*	A	A	None	S
111	*M. avium*	S	0.125	*M. avium* subsp. *hominissuis*	A	A	None	S
112	*M. avium*	S	1	*M. avium* subsp. *hominissuis*	A	A	None	S
113	*M. avium*	S	2	*M. avium* subsp. *hominissuis*	A	A	None	S
114	*M. avium*	I	16	*M. avium* subsp. *hominissuis*	A	A	None	S
115	*M. avium*	S	0.25	*M. avium* subsp. *hominissuis*	A	A	None	S
116	*M. avium*	S	1	*M. avium* subsp. *hominissuis*	A	A	None	S
2	*M. intracellulare*	S	0.25	*M. intracellulare* subsp. *intracellulare*	A	A	None	S
4	*M. intracellulare*	S	0.25	*M. intracellulare* subsp. *intracellulare*	A	A	None	S
7	*M. intracellulare*	S	0.25	*M. intracellulare* subsp. *intracellulare*	A	A	None	S
12	*M. intracellulare*	R	>32	*M. intracellulare* subsp. *intracellulare*	C	A	None	R
14	*M. intracellulare*	S	0.125	*M. intracellulare* subsp. *intracellulare*	A	A	None	S
15	*M. intracellulare*	S	0.125	*M. intracellulare* subsp. *intracellulare*	A	A	None	S
20	*M. intracellulare*	I	16	*M. intracellulare* subsp. *intracellulare*	A	A	None	S

29	*M. intracellulare*	R	32	*M. intracellulare* subsp. *intracellulare*	A	G	None	R
30	*M. intracellulare*	S	0.125	*M. intracellulare* subsp. *intracellulare*	A	A	None	S
31	*M. intracellulare*	S	0.25	*M. intracellulare* subsp. *intracellulare*	A	A	None	S
39	*M. intracellulare*	S	0.125	*M. intracellulare* subsp. *intracellulare*	A	A	None	S
50	*M. intracellulare*	S	0.125	*M. intracellulare* subsp. *intracellulare*	A	A	None	S
51	*M. intracellulare*	S	1	*M. intracellulare* subsp. *intracellulare*	A	A	None	S
59	*M. intracellulare*	S	0.25	*M. intracellulare* subsp. *intracellulare*	A	A	None	S
76	*M. intracellulare*	S	0.25	*M. intracellulare* subsp. *intracellulare*	A	A	None	S
78	*M. intracellulare*	S	0.25	*M. intracellulare* subsp. *intracellulare*	A	A	None	S
80	*M. intracellulare*	S	1	*M. intracellulare* subsp. *intracellulare*	A	A	None	S
84	*M. intracellulare*	S	0.5	*M. intracellulare* subsp. *intracellulare*	A	A	None	S
87	*M. intracellulare*	S	0.06	*M. intracellulare* subsp. *intracellulare*	A	A	None	S
89	*M. intracellulare*	S	0.25	*M. intracellulare* subsp. *intracellulare*	A	A	None	S
91	*M. intracellulare*	S	0.125	*M. intracellulare* subsp. *intracellulare*	A	A	None	S
95	*M. intracellulare*	S	0.06	*M. intracellulare* subsp. *intracellulare*	A	A	None	S
96	*M. intracellulare*	S	0.25	*M. intracellulare* subsp. *intracellulare*	A	A	None	S
107	*M. intracellulare*	S	1	*M. intracellulare* subsp. *intracellulare*	A	A	None	S

110	*M. intracellulare*	R	>32	*M. intracellulare* subsp. *intracellulare*	C	A	None	R
8	*M. intracellulare*	R	>32	*M. intracellulare* subsp. *chimaera*	A	C	None	R
10	*M. intracellulare*	S	2	*M. intracellulare* subsp. *chimaera*	A	A	None	S
17	*M. kansasii*	N.A.		*M. kansasii*	A	A	None	N.A.
43	*M. paragordonae*	N.A.		*M. paragordonae*	A	A	None	N.A.
85	*M. gordonae*	N.A.		*M. paragordonae*	A	A	None	N.A.
93	Unidentified	N.A.		*M. paragordonae*	A	A	None	N.A.
18	*M.lentiflavum*	N.A.		*M. lentiflavum*	A	A	None	N.A.
62	*M.lentiflavum*	N.A.		*M. lentiflavum*	A	A	None	N.A.
53	Unidenfitied	N.A.		*M. gordonae*	A	A	None	N.A.
73	*M.fortuitum complex*	N.A.		*M. fortuitum* subsp. *fortuitum*	A	A	None	N.A.
74	*M.peregrinum*	N.A.		*M.peregrinum*	A	A	None	N.A.
106	*M.peregrinum*	N.A.		*M.peregrinum*	A	A	None	N.A.
99	*M.chelonae*	N.A.		*M.chelonae*	A	A	None	N.A.
69	*M.fortuitum complex*	N.A.		*M.porcinum*	A	A	None	N.A.
71	*Unidentified*	N.A.		*M.szulgai*	A	A	None	N.A.

TRC, transcription-reverse transcription concerted reaction; MALDI-TOF MS, Matrix-assisted laser desorption ionization-time of flight mass spectrometry; PCR, polymerase chain reaction; R, resistant; S, susceptible; *M. abscessus, mycobacterium abscessus*; subsp, subspecies; N.A., not applicable.

Table S6. Efficacy of amikacin resistance prediction by MGIT-seq in comparison of drug susceptibility tests.

No.	*MLSTverse*	Drug susceptibility test		Amikacin resistance prediction by MinION	
				rrs position	Predicted amikacin susceptibility
		AMK	MIC	1408	
32	*M. abscessus* subsp. *abscessus*	S	8	A	S
72	*M. abscessus* subsp. *abscessus*	S	1	A	S
102	*M. abscessus* subsp. *abscessus*	S	4	A	S
19	*M. abscessus* subsp. *massiliense*	S	2	A	S
33	*M. abscessus* subsp. *massiliense*	S	8	A	S
44	*M. abscessus* subsp. *massiliense*	S	0.5	A	S
90	*M. abscessus* subsp. *massiliense*	S	16	A	S
103	*M. abscessus* subsp. *massiliense*	S	0.5	A	S
1	*M. avium* subsp. *hominissuis*	S	16	A	S
3	*M. avium* subsp. *hominissuis*	S	16	A	S
5	*M. avium* subsp. *hominissuis*	I	32	A	S
6	*M. avium* subsp. *hominissuis*	R	64	A	S
9	*M. avium* subsp. *hominissuis*	S	2	A	S
11	*M. avium* subsp. *hominissuis*	S	16	A	S
13	*M. avium* subsp. *hominissuis*	S	16	A	S
16	*M. avium* subsp. *hominissuis*	S	2	A	S
21	*M. avium* subsp. *hominissuis*	S	8	A	S
22	*M. avium* subsp. *hominissuis*	S	16	A	S
23	*M. avium* subsp. *hominissuis*	S	16	A	S

24	*M. avium* subsp. *hominissuis*	S	16	A	S
25	*M. avium* subsp. *hominissuis*	S	4	A	S
26	*M. avium* subsp. *hominissuis*	S	16	A	S
27	*M. avium* subsp. *hominissuis*	S	16	A	S
28	*M. avium* subsp. *hominissuis*	S	8	A	S
34	*M. avium* subsp. *hominissuis*	S	8	A	S
35	*M. avium* subsp. *hominissuis*	S	8	A	S
36	*M. avium* subsp. *hominissuis*	S	8	A	S
37	*M. avium* subsp. *hominissuis*	I	32	A	S
38	*M. avium* subsp. *hominissuis*	S	16	A	S
40	*M. avium* subsp. *hominissuis*	S	4	A	S
41	*M. avium* subsp. *hominissuis*	S	16	A	S
42	*M. avium* subsp. *hominissuis*	S	8	A	S
45	*M. avium* subsp. *hominissuis*	S	16	A	S
46	*M. avium* subsp. *hominissuis*	S	16	A	S
47	*M. avium* subsp. *hominissuis*	S	4	A	S
48	*M. avium* subsp. *hominissuis*	S	16	A	S
49	*M. avium* subsp. *hominissuis*	S	8	A	S
52	*M. avium* subsp. *hominissuis*	S	4	A	S
54	*M. avium* subsp. *hominissuis*	S	8	A	S
55	*M. avium* subsp. *hominissuis*	S	8	A	S
56	*M. avium* subsp. *hominissuis*	S	16	A	S
57	*M. avium* subsp. *hominissuis*	S	8	A	S

58	*M. avium* subsp. *hominissuis*	S	8	A	S
60	*M. avium* subsp. *hominissuis*	S	8	A	S
61	*M. avium* subsp. *hominissuis*	S	16	A	S
63	*M. avium* subsp. *hominissuis*	S	16	A	S
64	*M. avium* subsp. *hominissuis*	S	4	A	S
65	*M. avium* subsp. *hominissuis*	S	16	A	S
66	*M. avium* subsp. *hominissuis*	S	16	A	S
67	*M. avium* subsp. *hominissuis*	S	2	A	S
68	*M. avium* subsp. *hominissuis*	S	8	A	S
70	*M. avium* subsp. *hominissuis*	S	4	A	S
75	*M. avium* subsp. *hominissuis*	S	8	A	S
77	*M. avium* subsp. *hominissuis*	S	4	A	S
79	*M. avium* subsp. *hominissuis*	R	>256	G	R
81	*M. avium* subsp. *hominissuis*	S	8	A	S
82	*M. avium* subsp. *hominissuis*	S	16	A	S
83	*M. avium* subsp. *hominissuis*	S	8	A	S
86	*M. avium* subsp. *hominissuis*	S	1	A	S
88	*M. avium* subsp. *hominissuis*	S	8	A	S
92	*M. avium* subsp. *hominissuis*	S	8	A	S
94	*M. avium* subsp. *hominissuis*	S	1	A	S
97	*M. avium* subsp. *hominissuis*	S	8	A	S
98	*M. avium* subsp. *hominissuis*	S	4	A	S
100	*M. avium* subsp. *hominissuis*	S	4	A	S

101	*M. avium* subsp. *hominissuis*	S	16	A	S
104	*M. avium* subsp. *hominissuis*	S	8	A	S
105	*M. avium* subsp. *hominissuis*	S	8	A	S
108	*M. avium* subsp. *hominissuis*	S	4	A	S
109	*M. avium* subsp. *hominissuis*	S	8	A	S
111	*M. avium* subsp. *hominissuis*	S	4	A	S
112	*M. avium* subsp. *hominissuis*	S	8	A	S
113	*M. avium* subsp. *hominissuis*	S	8	A	S
114	*M. avium* subsp. *hominissuis*	S	4	A	S
115	*M. avium* subsp. *hominissuis*	I	32	A	S
116	*M. avium* subsp. *hominissuis*	S	2	A	S
2	*M. intracellulare* subsp. *intracellulare*	S	8	A	S
4	*M. intracellulare* subsp. *intracellulare*	S	4	A	S
7	*M. intracellulare* subsp. *intracellulare*	S	4	A	S
12	*M. intracellulare* subsp. *intracellulare*	S	2	A	S
14	*M. intracellulare* subsp. *intracellulare*	S	8	A	S
15	*M. intracellulare* subsp. *intracellulare*	S	8	A	S
20	*M. intracellulare* subsp. *intracellulare*	S	4	A	S
29	*M. intracellulare* subsp. *intracellulare*	S	8	A	S
30	*M. intracellulare* subsp. *intracellulare*	S	4	A	S
31	*M. intracellulare* subsp. *intracellulare*	S	4	A	S
39	*M. intracellulare* subsp. *intracellulare*	S	4	A	S
50	*M. intracellulare* subsp. *intracellulare*	S	4	A	S

51	*M. intracellulare* subsp. *intracellulare*	S	8	A	S
59	*M. intracellulare* subsp. *intracellulare*	S	8	A	S
76	*M. intracellulare* subsp. *intracellulare*	S	8	A	S
78	*M. intracellulare* subsp. *intracellulare*	S	8	A	S
80	*M. intracellulare* subsp. *intracellulare*	S	8	A	S
84	*M. intracellulare* subsp. *intracellulare*	S	8	A	S
87	*M. intracellulare* subsp. *intracellulare*	S	2	A	S
89	*M. intracellulare* subsp. *intracellulare*	S	4	A	S
91	*M. intracellulare* subsp. *intracellulare*	S	2	A	S
95	*M. intracellulare* subsp. *intracellulare*	S	2	A	S
96	*M. intracellulare* subsp. *intracellulare*	S	4	A	S
107	*M. intracellulare* subsp. *intracellulare*	S	4	A	S
110	*M. intracellulare* subsp. *intracellulare*	S	4	A	S
8	*M. intracellulare* subsp. *chimaera*	S	8	A	S
10	*M. intracellulare* subsp. *chimaera*	S	8	A	S
17	*M. kansasii*	N.A.			
43	*M. paragordonae*	N.A.			
85	*M. paragordonae*	N.A.			
93	*M. paragordonae*	N.A.			
18	*M. lentiflavum*	N.A.			
62	*M. lentiflavum*	N.A.			
53	*M. gordonae*	N.A.			
73	*M. fortuitum* subsp. *fortuitum*	N.A.			

74	*M.peregrinum*	N.A.
106	*M.peregrinum*	N.A.
99	*M.chelonae*	N.A.
69	*M.porcinum*	N.A.
71	*M.szulgai*	N.A.

TRC, transcription-reverse transcription concerted reaction; MALDI-TOF MS, Matrix-assisted laser desorption ionization-time of flight mass spectrometry; PCR, polymerase chain reaction; R, resistant; I, intermediate; S, susceptible; *M. abscessus, mycobacterium abscessus*; subsp, subspecies; N.A., not applicable.

Table S7. Costs for direct MGIT sequence

Cost for Library preparation

Item	Cost	Consumption				Additional Information
		DNA	Library	Sequencing	Total	Product
P1000 pipette tips	$0.20			2	2	One Touch Filter tips 1000 µL
P200 pipette tips	$0.80	4	2	4	10	One Touch Filter tips 200 µL
P10 pipette tips	$0.48	2	3	1	6	One Touch Filter tips 10 µL
1.5 ml Eppendorf DNA LoBind tubes	$0.27	1	3		4	DNA LoBind Tubes
0.2 ml thin-walled PCR tubes	$1.33		1		1	0.2ml 8-Tube (q)PCR Strips, with Single Attached Caps, High Profile
Nuclease-free water	$0.001		20uL	4.5uL	25uL	DISTILLED WATER
Freshly prepared 70% ethanol in nuclease-free water	$0.010		500uL		500uL	99.5% Ethanol
10 mM Tris-HCl pH 8.0 with 50 mM NaCl	$0.002		10uL		10uL	1M Tris-HCl (pH 8.0)
Glass beads	$2.27	1			1	MN Bead Tubes Type B
Qubit dsDNA HS Assay	$1.36	1	1		2	Qubit dsDNA HS Assay Kit, 500 assays
Agencourt AMPure XP beads	$0.53		30uL		30uL	Agencourt AMPure XP
LongAmp Taq 2X Master Mix (e.g. NEB M0287)	$1.15		25uL		25uL	LongAmp Taq 2X Master Mix
Cost/sample for Library preparation	$8.40					

Total cost for sequencing run

Item	Cost
12 Prepared Libraries	$100.8
Rapid PCR Barcoding Kit (SQK-RPB004)	$108.3
Flow Cell (R9.4.1)	$500
Total cost / sample	$59.1

MGIT, mycobacterial growth indicator tube; PCR, polymerase chain reaction.
